# A Two‐Stage Method for Extending Inferences From a Collection of Trials

**DOI:** 10.1002/sim.70146

**Published:** 2025-06-05

**Authors:** Nicole Schnitzler, Eloise Kaizar

**Affiliations:** ^1^ Ohio Colleges of Medicine Government Resource Center The Ohio State University Columbus Ohio USA; ^2^ Department of Statistics The Ohio State University Columbus Ohio USA

**Keywords:** causal inference, generalizability, meta‐analysis, transportability

## Abstract

When considering the effect a treatment will cause in a population of interest, we often look to evidence from randomized controlled trials. In settings where multiple trials on a treatment are available, we may wish to synthesize the trials' participant data to obtain causally interpretable estimates of the average treatment effect in a specific target population. Traditional meta‐analytic approaches to synthesizing data from multiple studies estimate the average effect among the studies. The resulting estimate is often not causally interpretable in any population, much less a particular target population, due to heterogeneity in the effect of treatment across studies. Inspired by traditional two‐stage meta‐analytic methods and methods for extending inferences from a single study, we propose a two‐stage approach to extending inferences from a collection of randomized controlled trials that can be used to obtain causally interpretable estimates of treatment effects in a target population when there is between‐study heterogeneity in conditional average treatment effects. We first introduce a collection of assumptions under which the target population's average treatment effect is identifiable when conditional average treatment effects are heterogeneous across studies. We then introduce an estimator that utilizes weighting in two stages, taking a weighted average of study‐specific estimates of the treatment effect in the target population. We assess the performance of our proposed approach through simulation studies and two applications: A multi‐center randomized clinical trial studying a Hepatitis‐C treatment and a collection of studies on a therapy treatment for symptoms of pediatric traumatic brain injury.

## 1 Introduction

Policymakers, clinicians, and others are often interested in learning what effect a treatment will have on the populations they serve. But it is typically infeasible and potentially unethical to conduct internally valid randomized controlled trials (RCTs) whose participants represent every target population of interest. A growing collection of work addresses the limited natural extensions of RCT results to specific target populations due to treatment effect heterogeneity [1, 2, 3, 4]. When data are available, jointly analyzing an entire collection of relevant RCTs is likely to provide the best available information about the treatment effect in a target population. Traditional approaches to individual participant data (IPD) meta‐analysis are designed to obtain an estimate among the population of study participants. Therefore, resulting estimates are often not causally interpretable in any meaningful population [5]. We propose a framework that generalizes IPD meta‐analytic methods and allows us to extend inferences from an existing collection of studies to obtain causally interpretable estimates of treatment effects in a specific target population. We follow Dahabreh and Hernán [6] to use the general term “extension” to refer to making inference about a counterfactual quantity (in our case, the average treatment effect) for a specific population.

Traditional two‐stage approaches to IPD meta‐analysis synthesize information from collections of studies on the same treatment to obtain a single effect estimate. In the first stage, researchers obtain separate effect estimates from each study. These estimates are then combined in the second stage, often via a weighted average [7, 8]. Two‐stage approaches are generally framed as either a fixed effect (FE) approach, consistent with the notion of a common true effect size across all of the studies, or a random effect (RE) approach, consistent with an assumption that the true study effects are drawn from some distribution [8, 9]. We also follow the two‐stage approach, where we estimate causally interpretable effect sizes from each study in the first stage and incorporate study‐to‐study variability using an RE approach in the second stage.

For the first stage, we incorporate existing methods to extend inferences from a single study to a target population by using propensity‐like weights. That is, we weight the study data to emulate the distribution of target population characteristics. We do this by repurposing the potential outcomes framework for causal inference proposed by Rubin [10], considering an individual's potential outcome under one treatment to be the outcome that would be observed if the individual received that treatment. A study observation will typically only be observed under one treatment, so the potential outcomes under all other available treatments are missing. The traditional concern of causal inference is selection bias due to these structurally missing potential outcomes across the observed control and treatment groups. In our extension setting, we are concerned with structurally missing observed potential outcomes in the target population. Our goal is to estimate the features of the distribution of potential outcomes among individuals in a specific target population, but these individuals were never enrolled in a study or assigned to a treatment. In this article, we focus on methods for extending inferences to a target population that has no overlap with the populations of study participants. This type of extension is often referred to as transportation (as opposed to generalization), but there has yet to be broad agreement on terminology [4, 6, 11].

A collection of researchers recently used this framework to extend inferences from a collection of studies without explicitly addressing study‐to‐study heterogeneity that remains after adjusting for measured covariates. As discussed in more detail in later sections, these methods fully or partially pool studies' IPD, in a sense treating the data as though it had come from one large study instead of a collection of smaller ones. These methods are consistent with the assumption that participation in a particular study is not associated with an individual's expected treatment effect, conditional on observed covariates [6, 12, 13]. Under this assumption, an individual's expected treatment effect, conditional on their covariates, would not change if they move from the target population to a study or from one study to another. In settings where there are between‐study differences in research group, environment, trial design, etc., methods based on this assumption may not capture key features of the true treatment effect distributions. Vo et al. [14, 15] explores such heterogeneity for case‐mix‐adjusted meta‐analysis. Our proposed approach incorporates such heterogeneity in the extending inferences setting, thus providing high‐quality inference for a broader range of collections of studies by allowing for between‐study treatment effect heterogeneity beyond that explained by measured covariates. This early phase work relaxes the key assumption, mentioned above, about the conditional relationship between the treatment effect in the target population and in each of the studies (see the discussion of Assumption B4 in Section 4.1), and we plan to, in future work, embrace an even wider range of realistic distributional characteristics for the random effect.

To motivate our work, we consider two real data applications. The first extends the results of a multi‐center trial of a treatment for cirrhosis to a target population that somewhat mimics the United States population of patients who would meet the treatment criteria. We treat each center separately in the first stage of analysis and expect identical inclusion criteria, protocol, procedures, etc. across the centers to result in relatively homogeneous treatment effects. In contrast, our second example is a meta‐analysis of a collection of clinical trials studying the impact of an online therapy treatment on symptoms of pediatric traumatic brain injury (TBI). Because these studies were conducted by different research groups, at different times, utilizing different enrollment sites, and employing different eligibility criteria, we anticipate study‐level heterogeneity in treatment effects.

The rest of this article is organized as follows. We first introduce relevant notation and inferential targets in Section 2. Weighted methods for extending inferences that pool studies' IPD are reviewed in Section 3. Then, in Section 4, we introduce a two‐stage approach that allows for between‐study heterogeneity in treatment effects. We consider the performance of approaches to extending inferences from a collection of studies using simulation studies in Section 5. Applications to our motivating examples are presented in Section 6. Finally, Section 7 summarizes our work, discusses its implications, and considers potential areas of future work. Notably, our discussion highlights the preliminary nature of this work, akin to a “Phase I” methodological development [16]. Future work highlights the adaptations we anticipate will allow us to relax the strong assumptions outlined in Section 4, to better align with real‐world applications.

## 2 Notation and Inferential Targets

We assume that we have IPD from a collection of m RCTs. Each tests a pair of treatments $𝒜=\{a,a^{\prime }\}$ where a represents the treatment and $a^{\prime }$ represents the control. In addition, we assume we have a simple random sample (SRS) of individuals from an infinite target population of interest that does not include any study participants, for which we have measured all baseline covariates but no treatments or outcomes. Across the combined RCT and population data, we have measurements on n individuals indexed by i=1,2,…,n. Let $S_{i}\in \{0,𝒮\}$ be a random variable denoting the study membership for observation i with $S_{i}=0$ denoting membership in the target population. Then, $n_{s}=\sum_{i=1}^{n}I(S_{i}=s)$ for s∈{0,𝒮} is the sample size for each data source and $n=\sum_{s=0}^{m}n_{s}$. Throughout this article, we use the notation f(·) to denote densities.

For each RCT study participant $i:S_{i}\neq 0$, we observe variables including a study indicator, $S_{i}$, treatment assignment, $A_{i}$, an outcome of interest, $Y_{i}$, and a vector of baseline covariates, $X_{i}$. Within each RCT, these vectors are mutually independent and identically distributed. Because observations in our target population are not enrolled in a study, for each observation in our sample from the target population, we observe only the study indicator $S_{i}=0$ and a vector of baseline covariates $X_{i}$.

To aid our focus on estimating causal effects, we adopt the notation of Rubin [10] and let $Y_{i}^{a}$ be the potential outcome for observation i under treatment a. This is the outcome that would be observed for observation i if they received treatment a. We focus on two common inferential targets when extending inferences: (1) the target population's average treatment effect (TATE),

(1) $$\Delta =E\left(Y_{i}^{a}-Y_{i}^{a^{\prime }}|S_{i}=0\right)$$

that is, the expected treatment effect for a randomly selected individual in the target population, and (2) the mean potential outcome under a∈𝒜 in the target population, $\mu_{a}=E\left(Y_{i}^{a}|S_{i}=0\right)$, that is, the expected outcome under treatment a∈𝒜 for a randomly selected individual in the target population. Notice that $\Delta =\mu_{a}-\mu_{a^{\prime }}$, so estimators of Δ can often be viewed as differences between estimates of mean potential outcomes.

## 3 Existing Methods for Extending Inferences

Recently, methods for extending inferences that can be thought of as fully or partially pooling studies' IPD have been proposed [12, 13]. By effectively pooling the study data (after accounting for potentially different treatment assignment strategies), these methods, in a sense, treat the data as though they had come from one large study instead of a collection of smaller ones. Versions of these methods include approaches that rely on modeling the conditional average treatment effect (ATE) in the pooled study population, that is, for $i:S_{i}\in 𝒮$, (e.g., see Dahabreh et al. [12, 13]) and adaptations for settings where covariate data are systematically missing from some of the studies in 𝒮 (e.g., see Steingrimsson et al. [17]). Such methods could build on weighted, model‐based, or doubly‐robust/augmented methods for extending inferences from a single study. In this article, we focus on weighted methods for estimating the TATE in an infinite target population.

### 3.1 Assumptions

When extending inferences, we must make a collection of assumptions that enable us to obtain causally interpretable estimates from the study data and to extend those estimates to our target population. Such assumptions could be very strong. For example, assumptions of homogeneous treatment effects and selection into studies along with an assumption of treatments that mimic simple random sampling would result in most naïve methods yielding valid causally interpretable inference. Much modern research in causal inference adopts a set of much weaker assumptions. The specifics may vary by the inferential target and method of estimation, but approaches generally involve a collection of one assumption from each of five categories.

The first three categories consist of variations of the usual causal inference assumptions about the consistency and exchangeability of potential outcomes and the positivity of treatment assignment. Dahabreh et al. [12, 13] propose the following assumptions for these categories:

**A1**. If $A_{i}=a$, then $Y_{i}^{a}=Y_{i}$ for all i=1,…,n and a∈𝒜.
**A2**. For all s∈𝒮, for all a∈𝒜, and for x such that f(x,S=s)>0,
$$E\left(Y^{a}|X=x,S=s,A=a\right)=E\left(Y^{a}|X=x,S=s\right).$$
**A3**. For all s∈𝒮, for all a∈𝒜, and for x such that f(x,S=s)>0,
P(A=a|X=x,S=s)>0.

Assumption A1 implies consistency of the potential outcomes regardless of study membership. Exchangeability of the potential outcomes is implied by Assumption A2, which states that, conditional on their baseline covariates, an individual's mean potential outcome in a given study does not depend on their treatment assignment. Finally, Assumption A3 states that within each study, there are no sets of possible baseline covariates that would prevent an individual from receiving any of the treatments being considered.

The fourth and fifth categories are exchangeability and positivity assumptions to enable extension from the studies to the target population. Dahabreh et al. [12, 13] propose the following:

**A4**. For all s∈𝒮, for all $a,a^{\prime }\in 𝒜$ and for x such that f(x,S=0)>0,
$$E\left(Y^{a}-Y^{a^{\prime }}|X=x,S=0\right)=E\left(Y^{a}-Y^{a^{\prime }}|X=x,S=s\right).$$
**A5**. For all s∈𝒮 and for x such that f(x,S=0)>0,
P(S=s|X=x)>0.

Assumption A4 states that individuals' ATEs, conditional on their covariate values, are the same across all of the studies and the target population. Under this assumption, an individual's treatment effect is conditionally independent of their study membership. This implies that study membership does not need to be accounted for except to allow for differences in the treatment assignment mechanism across studies. Therefore, data from the RCTs can be pooled (after accounting for treatment assignment) when extending inferences if this assumption holds. Assumption A5 requires sufficient overlap between the covariate distribution in the target population and each individual study. When the study data are pooled, Dahabreh et al. note that we can relax this assumption and only require sufficient overlap between the target population and the pooled study data [12, 13].

### 3.2 Weighted Estimators

One type of existing estimator of the TATE based on observations from the (partially) pooled study sample is a weighted average. These estimators take the form

(2) $$\hat{\Delta }=\overset{n}{\underset{i=1}{\sum }}\left(\frac{{ŵ}_{i}(a)}{\sum_{i=1}^{n}{ŵ}_{i}(a)}-\frac{{ŵ}_{i}(a^{\prime })}{\sum_{i=1}^{n}{ŵ}_{i}(a^{\prime })}\right)Y_{i}$$

where, for each a∈𝒜, ${ŵ}_{i}(a)\geq 0$ with ${ŵ}_{i}(a)=0$ if $S_{i}=0$ or $A_{i}\neq a$. It is easy to see that $\hat{\Delta }$ is the difference between weighted averages of the treated and control units in the study sample, which could be used separately to estimate $\mu_{a}$ and $\mu_{a^{\prime }}$. Because existing weighted methods (partially) pool the study data, the estimator of TATE in Equation (2) has the same form as estimators proposed for extension from a single study (e.g., see Buchanan et al. [1], Dahabreh et al. [6]). Specific estimators are differentiated by different choices for the estimated weights, ${\left\{{ŵ}_{i}(\cdot )\right\}}_{i=1}^{n}$.

A naïve approach sets the estimated weights to be a simple indicator of receiving treatment in any study

(3) $${ŵ}_{ua,i}\left(a\right)=I\left(A_{i}=a\right)I\left(S_{i}\neq 0\right)$$

for each a∈𝒜, where ua stands for unadjusted. The resulting estimator, ${\hat{\Delta }}_{ua}$, is the difference between the average outcomes among the treated units across all studies and the control units across all studies. These unadjusted weights are appealing because of their simplicity, but ${\hat{\Delta }}_{ua}$ does not adjust for potential confounding due to treatment assignment or study membership. Therefore, ${\hat{\Delta }}_{ua}$ will only be a consistent estimator of the TATE in very special cases such as when the pooled study data is a SRS from the target population, potential outcomes are consistent and independent of both treatment assignment and study, and treatment assignment is independent of both baseline covariates and study membership.

Dahabreh et al. [12] adjust for potential confounding via estimated weights for each a∈𝒜:

(4) $${ŵ}_{pool,i}\left(a\right)=\left(\frac{I\left(A_{i}=a\right)}{{ê}_{a}\left(X_{i},S_{i}\right)}\right)\left(\frac{\hat{p}\left(X_{i},0\right)}{1-\hat{p}\left(X_{i},0\right)}\right)I\left(S_{i}\neq 0\right)$$

where $\hat{p}\left(X_{i},0\right)$ and ${ê}_{a}\left(X_{i},S_{i}\right)$ are estimates of $P\left(S=0|X_{i}\right)$ and $P\left(A=a|X_{i},S_{i}\right)$, respectively. The first fraction in Equation ((4)) is the inverse‐probability of treatment weight (IPTW), which adjusts for potential confounding due to treatment assignment. The second fraction is the inverse odds of study participation weight (IOSPW), which adjusts for potential confounding due to study membership. Under Assumptions A1 through A5, ${\hat{\Delta }}_{pool}$, is a consistent estimator of the TATE so long as $\hat{p}(X,0)$ and ${ê}_{a}(X,S)$, for each a∈𝒜, are consistent estimators of corresponding probabilities [12, 13].

A more recently proposed method for causal interpretable meta‐analysis does not require pooling study data [18]. This proposed method utilizes the same estimator as we propose in Section 4.2, but considers the distinct estimand of the expected treatment effect we would see *if we could run a new trial in the target population*. They do this by conceptualizing a two‐dimensional intervention consisting of both treatment and study and following the lead of VanderWeele and Hernán [19] to consider a two‐dimensional potential outcome. Considering the study akin to a treatment variation (rather than a covariate, as we propose) leads to assumptions and operating characteristics that differ from our proposal [18].

## 4 A Two‐Stage Approach to Extending Inferences

### 4.1 Assumptions

We first choose a set of assumptions that span the five categories introduced in Section 3.1. We directly adopt three of the assumptions proposed by Dahabreh et al. [12, 13]: A2, A3, and A5. Assumptions A1 and A4 restrict inferences to settings where individuals' potential outcomes and conditional ATEs are not associated with the study, so we propose alternative assumptions for these categories.

We relax the consistency Assumption A1 to allow an individual's potential outcomes to differ across different study participation, even if all their measured pre‐treatment covariates remained the same. It is easy to imagine relevant real‐world situations. For example, consider two RCTs of the same treatment that were conducted at hospital systems in two different communities. We might expect to see better outcomes among some patients in one hospital than in the other because of differences in unmeasured community and hospital‐level characteristics. In particular, we assume:

**B1**. For all s∈𝒮, a∈𝒜, and $i\in \{1,2,\ldots ,n:S_{i}=s\}$, if $A_{i}=a$ then $Y_{i}^{a}=Y_{i}$.

Note that we do not specify any relationship between the potential and observed outcomes in the target population (s=0). Such specification is unnecessary because we do not observe outcomes for members of the target population.

We further relax the exchangeability Assumption A4 to allow for study‐level treatment effect heterogeneity due to unmeasured covariates. We assume only that the average of the conditional ATEs follows some distribution with a mean at the target population's conditional ATE. Note that this distribution across the studies is not conditional on the covariates, and so it is not simply an application of iterated expectations. Instead, we are averaging across the same single distribution for every possible fixed vector of covariates. In the language of “do‐calculus” [20], we are averaging the conditional effect size given do(x) (fixing the value of x and thus eliminating the joint distribution of S and X) over the distribution of studies. The resulting assumption is:

**B4**. For all $a,a^{\prime }\in 𝒜$ and for x such that f(x,S=0)>0,
(5) $$\begin{array}{cc} & E\left(\left.Y^{a}-Y^{a^{\prime }}\right|X=x,S=0\right)\\ & \;=E_{S}\left[E\left(\left.Y^{a}-Y^{a^{\prime }}\right|X=x,S\right)|.S\in S\right]. \end{array}$$

Note that Assumption A4 is sufficient but not necessary for Assumption B4 to hold. A4 implies that the object of the expectation inside the square brackets in Equation ((5)) would be constant and thus the outer expected value would be extraneous. Supposing that

$$E\left(Y^{a}-Y^{a^{\prime }}|X=x,S\right)=\left\{\begin{array}{ll} \frac{\left(m+1\right)}{2} & \text{for}\;S=0\\ S & \text{otherwise} \end{array}\right.$$

provides a clear (though silly) counterexample for necessity if S follows a discrete uniform distribution.

We generally see Assumption B4 as moving the traditional meta‐analysis symmetric random effect distribution assumption from the study level to the individual conditional level and applying it within the counterfactual framework. Under Assumption B4, our defined estimand of interest in Equation (1) is identical to the center of the multivariate distribution of study‐specific conditional average effect sizes, averaged over a pre‐specified target distribution of covariates X. Somewhat parallel to the development of traditional meta‐analysis, we plan to further relax this assumption to account for more realistic data production and collection situations in future work.

Our final collection of identifiability assumptions consists of assumptions B1, A2, A3, B4, and A5. Moving forward, we will call this set of assumptions “Assumption ℬ” for short.

#### 4.1.1 Identifiability

When we can write the TATE in terms of features that can be estimated from observed data, we say that the TATE is identifiable. Under Assumption ℬ and a regularity condition, the TATE, as defined in Equation (1), is identifiable.

Theorem 1 *Under the regularity condition*

$$E_{S}\left\{E_{X}\left[\left.|E_{Y^{a},Y^{a^{\prime }}}\left(Y^{a}-Y^{a^{\prime }}|X,S\right)|\;\right|S=0\right].|S\in S\right\}<\infty$$

*and Assumption* ℬ,

(6) $$\Delta \equiv E(Y^{a}-Y^{a^{\prime }}|S=0)=E_{S}\left(\Delta_{S}|S\in 𝒮\right)$$

*with*

(7) $$\begin{array}{rl} \Delta_{s}\equiv & \;E_{X}\left[E_{Y^{a},Y^{a^{\prime }}}\left(\left.Y^{a}-Y^{a^{\prime }}\right|X,S=s\right).|S=0\right]\\ = & \;E_{A,X,Y}\left(\left.\left[\frac{w(a,s)}{E_{A,X}\left(w(a,s)|S=s\right)}-\frac{w(a^{\prime },s)}{E_{A,X}(w(a^{\prime },s)|S=s)}\right]Y\right|S=s\right) \end{array}$$

*for each* s∈𝒮 *where*

$$w(a,s)=\frac{I(A=a)}{P(A=a|X,S=s)}\frac{P(S=0|X)}{P(S=s|X)}.$$

*for each* s∈𝒮 *and* a∈𝒜.

A detailed proof of this result can be found in Appendix A.

### 4.2 Estimator

Based on the identifiability result in Equation (6), we propose a class of two‐stage estimators for the TATE:

(8) $${\hat{\Delta }}_{two}=\frac{1}{\sum_{s=1}^{m}w_{s}}\overset{m}{\underset{s=1}{\sum }}w_{s}{\hat{\Delta }}_{s}$$

where ${\left\{w_{s}\right\}}_{s\in 𝒮}$ are strictly positive study‐level weights and ${\left\{{\hat{\Delta }}_{s}\right\}}_{s\in 𝒮}$ are study‐specific estimates of the TATE. This estimator is similar to the weighted estimator typically used in two‐stage IPD meta‐analyses, but has one key difference. Unlike these more traditional meta‐analytic approaches, ${\hat{\Delta }}_{two}$ utilizes causally interpretable study‐specific estimates of the effect of a treatment in a specific target population.

Based on the identifiability result in Equation (7), we propose utilizing the study‐specific weighted estimator of the TATE for each study s∈𝒮:

(9) $${\hat{\Delta }}_{s}=\underset{i:S_{i}=s}{\sum }\left(\frac{{ŵ}_{i}\left(a,s\right)}{\sum_{i:S_{i}=s}{ŵ}_{i}\left(a,s\right)}-\frac{{ŵ}_{i}\left(a^{\prime },s\right)}{\sum_{i:S_{i}=s}{ŵ}_{i}\left(a^{\prime },s\right)}\right)Y_{i}$$

where

(10) $${ŵ}_{i}\left(a,s\right)=\left(\frac{I\left(A_{i}=a\right)}{{ê}_{a}\left(X_{i},s\right)}\right)\left(\frac{\hat{p}\left(X_{i},0\right)}{\hat{p}\left(X_{i},s\right)}\right)$$

$e_{a}\left(x,s\right)=P\left(A=a|X=x,S=s\right)$, and p(x,s)=P(S=s|X=x) for each a∈𝒜 and s∈𝒮 with hats indicating estimates of these quantities. This study‐specific estimator has a similar form to the pooled weighted estimator, ${\hat{\Delta }}_{pool}$, weighting each observation in study s by a study‐specific IPTW and the inverse of its odds of being enrolled in the study as opposed to the target population. Each member of this class of estimators is distinguished by: (a) the methods used to estimate the study‐specific propensities for treatment assignment and study membership and (b) the specification of the study‐level weights.

For (a), we simplify our presentation and theoretical derivations by using parametric binomial and multinomial logistic regression models to estimate the study‐specific propensities. Similar approaches have been used in related prior work (e.g., Dahabreh et al. [6]). To allow for different treatment assignment mechanisms across studies, we propose fitting separate logistic regression models for treatment within each study to obtain estimates of each individual's propensity of treatment within their assigned study, $e_{a}(X_{i},S_{i})$, noting that $e_{a^{\prime }}(X_{i},S_{i})=1-e_{a}(X_{i},S_{i})$. To simultaneously estimate the conditional probabilities of study membership, p(x,s), s∈{0,𝒮}, we propose fitting a single multinomial logistic regression model for study membership, where membership in the target population is considered to be the baseline category. We do not consider random‐effect study‐specific or study membership models because the primary purpose of the unit‐level weights is to obtain balance [21]. In practice, one could choose some other parametric or nonparametric methods; exploration of these methods is left to future work.

For (b), we begin by considering desirable properties for the study‐level weights. We would prefer weights that produce a consistent estimator of the TATE, and conjecture that equal weights across the studies would achieve consistency. A detailed outline of our reasoning for this conjecture can be found in the Supporting Information. Other weighting schemes may be considered, such as a variance‐inspired weight to minimize the uncertainty in the TATE estimation, or an overlap‐inspired weight to ensure that studies with characteristics most similar to the target population are most influential. These (and other) alternative weighting schemes are left to future work.

#### 4.2.1 Variance Estimation

To obtain variance estimates and confidence intervals for ${\hat{\Delta }}_{two}$, we propose a stratified bootstrap procedure that mimics our imagined data‐generating procedure by resampling from the studies and the target population separately. We first take a bootstrap sample of size m from the target population sample by sampling with replacement and equal probabilities, maintaining the original sample size. Second, we obtain a bootstrap sample of the study data by following a two‐stage procedure. To capture study‐level heterogeneity in treatment effects, we first sample m studies with replacement and equal probability from 𝒮. Within each resampled study, we reflect the within‐study variability by taking separate bootstrap samples within each treatment arm, maintaining the sample sizes in the observed data so that we effectively maintain the observed designs for possibly different subsets of participants. Finally, we use the sample variance of the TATE estimates calculated for each bootstrap resample to estimate the variance of ${\hat{\Delta }}_{two}$ [22]. We use percentile intervals to obtain approximate confidence intervals (CIs) [23].

## 5 Simulation Studies

To examine the performance of our proposed two‐stage estimator of the TATE, we conducted proof‐of‐concept simulations. We attempted to capture a range of scenarios where the treatment effect heterogeneity across studies may have more or less influence on the ultimate inference. Simulations and summaries were conducted using R versions 3.5.1, 4.2.1, and 4.4.0 [24]. Logistic regression models were fit using the glm function in the stats base R package. Multinomial logistic regression models were fit using the vglm function in the VGAM package [25, 26], where estimate convergence was assessed directly on the key feature for our method—the model coefficient estimate values. The package rsimsum [27] was used to estimate bias, MSE, and EmpSE. Each simulation study included 1 000 replications, as described below.

Our simulation studies admittedly favor the proposed two‐stage estimator, since the data‐generating procedure meets all of the assumptions ℬ. In particular, we have set the mean of the distribution of the $\theta_{s}$ to be the vector of coefficients for the outcome generation model in the target population. In contrast, all of the assumptions outlined in Section 3.1 are not met, thus likely disfavoring the pooled estimator. We discuss our choices in more detail and in relation to future work in Section 7.

**Number and Size of Studies.** We considered collections of three, ten, and thirty studies, thus representing settings that span a small but realistic collection through a large collection where asymptotic properties may begin to emerge. For each size collection, we considered large and small studies. Large study simulations included approximately 5500 participants in the target population and 1500 per study, on average, whereas the small study simulations included only 200 participants in the target population and 100 per study, on average. Because we conjecture that estimator behavior may depend on the distribution of sample sizes across the studies, we consider a case where the study sizes are all approximately the same and cases where $n_{s}$ increases with s, with largest study between 4 and 10 times that of the smallest study. Average sizes are reported in more detail in the Supporting Information.

**Variable Distributions.** To bolster transparency, we chose to use only one covariate. Samples from the joint distribution among the covariate, study membership, and treatment were generated as follows.

1. For each individual $i=1,2,\ldots ,n\sum_{s=0}^{m}n_{s}$, independently draw covariate $X_{i}$ from a standard normal distribution.
2. For each individual $i=1,2,\ldots ,n\sum_{s=0}^{m}n_{s}$, independently assign $S_{i}\in \left\{0,\ldots ,m\right\}$ using a multinomial logistic regression model where, recalling that the expit function is the inverse of the logit function and noting the constraint that $\sum_{s=0}^{m}P\left(S_{i}=s|X_{i}\right)=1$,
(11) $$P\left(S_{i}=s|X_{i}\right)=\text{expit}\left(\beta_{s,0}+X_{i}\beta_{s,1}\right)\text{, for}\;s=1,2,\ldots ,m$$
  Values for the intercepts were chosen via a numeric algorithm [28] to achieve the desired sample size patterns discussed above. Values for the covariate coefficients were chosen to induce different conditional distributions of the covariate across the studies, with the center of the conditional distribution of X generally increasing from s=1 to s=m and the center of the conditional distribution of X for the target population generally centered at or above these. Exact values are reported in the Supporting Information.
3. For all individuals, each study s=1,…,m, independently assign a vector of treatments according to a completely randomized treatment assignment mechanism with $P\left(A_{i}=a|S_{i}=s\right)=0.5$.
4. For each study s=1,…,m, prepare to create treatment effect heterogeneity by generating a vector of four outcome model coefficients $\theta_{s}={\left(\nu_{s},\gamma_{s},\lambda_{s},\kappa_{s}\right)}^{t}$ from a distribution centered at $\theta_{0}$, the coefficients assumed for the target population:
$$\theta_{s}\overset{iid}{∼}N\left(\theta_{0}=\left(\begin{array}{c} -1\\ -1\\ 0.5\\ -0.5 \end{array}\right),\sum_{\theta }=\left(\begin{array}{cccc} 0.5 & 0 & 0 & 0\\ 0 & \sum_{\gamma } & 0 & 0\\ 0 & 0 & 0.5 & 0\\ 0 & 0 & 0 & \sum_{\kappa } \end{array}\right)\right).$$
5. Generate outcomes according to the outcome generation linear model:
(12) $$Y_{i}=\nu_{S_{i}}+A_{i}\gamma_{S_{i}}+X_{i}\lambda_{S_{i}}+A_{i}X_{i}\kappa_{S_{i}}+\epsilon_{i}$$
  where $\epsilon_{i}\overset{iid}{∼}N(0,1)$ are mutually independent of $X_{i}$, $A_{i}$, and $S_{i}$ for i=1,2,…,n.

Note that the true TATE is thus specified to be:

(13) $$E\left(Y^{1}-Y^{0}|S=0\right)=-1-0.5E\left(X|S=0\right)$$

and the study‐specific conditional ATEs for s∈{0,𝒮} are:

(14) $$E\left(Y^{1}-Y^{0}|S=s,X=x\right)=\gamma_{s}+\kappa_{s}x$$

We conjectured that estimator performance may vary according to the main source of across‐study treatment effect heterogeneity: (a) effects of measured covariates that vary across studies (as reflected by ${\left\{\kappa_{s}\right\}}_{s\in 𝒮}$ with large sample variance) or (b) effects of unmeasured covariates that vary across studies (as reflected by ${\left\{\gamma_{s}\right\}}_{s\in 𝒮}$ with large sample variance). For cases where the measured covariates dominate, we set $\sum_{\gamma }=0.1$ and $\sum_{\kappa }=2$; for cases where the unmeasured covariates dominate, we set $\sum_{\gamma }=2$ and $\sum_{\kappa }=0.1$. For each of the six combinations of collection and study sizes, we considered three combinations of study size distributions and main source of treatment effect heterogeneity, which are summarized in Table 1.

**TABLE 1:** General simulation settings considered for three, ten, and thirty small and large study collections.

		Main source of treatment
Setting	Study sizes	Effect heterogeneity
1	Similar	Measured features ($\sum_{\gamma }<\sum_{\kappa })$
2	Different	Measured features ($\sum_{\gamma }<\sum_{\kappa })$
3	Different	Unmeasured features ($\sum_{\gamma }>\sum_{\kappa })$

**Estimators and Evaluation.** We studied three estimators: The naïve unadjusted estimator implemented as in Equation (2) with weights as in Equation (3), the pooled estimator implemented as in Equation (2) with weights as in Equation (4), and our proposed two‐stage estimator implemented as in Equation (8) with equal weights across the studies. We estimate standard errors and confidence intervals via 1000 (or 500 for m=30) stratified bootstrap samples as proposed in Section 4.2.1, focusing this exploration on Setting 2. We evaluate point estimator performance via bias, mean squared error (MSE), and empirical standard error (EmpSE), and evaluate uncertainty estimation via CI coverage and relative error. To calculate the true TATE in each setting, we obtained empirical approximations of the expected value of X in the target population. Details can be found in the Supporting Information.

**Simulation Results** Select results from simulations with three, ten, and thirty large studies can be found in Table 2; see the Supporting Information for full results. Not surprisingly, the unadjusted estimator is biased towards the treatment effect in the largest of the m studies. In contrast, both the pooled and two‐stage estimators are essentially unbiased in all settings considered, leading to the EmpSE essentially determining the MSE. Under all settings considered, the two‐stage estimator had lower EmpSE than the existing pooled estimator. The lower variability for the two‐stage estimator is most pronounced in Setting 3, where the study sizes differed and unmeasured study features were the main source of differences in treatment effect heterogeneity across studies. The patterns for the point estimate results from simulations with smaller‐sized studies were similar and can be found in the Supporting Information.

**TABLE 2:** Results from simulations with m=3 large studies and select results from simulations with m=10 and m=30 largeg studies.

		Setting 1	Setting 2	Setting 3	Setting 2	Setting 2
Metric	Estimator	m=3	m=3	m=3	m=10	m=30
Bias	Unadjusted	0.091 (0.0080)	0.056 (0.0074)	0.099 (0.0286)	0.075 (0.0038)	0.058 (0.0023)
	Pooled	−**0.007 (0.0077)**	−**0.002 (0.0079)**	**0.040 (0.0287)**	**0.005 (0.0042)**	−**0.003 (0.0027)**
	Two‐stage	−**0.003 (0.0062)**	−**0.006 (0.0061)**	**0.035 (0.0256)**	**0.003 (0.0036)**	−**0.004 (0.0021)**
EmpSE	Unadjusted	0.252 (0.0056)	0.235 (0.0053)	0.903 (0.0202)	0.119 (0.0027)	0.073 (0.0016)
	Pooled	0.242 (0.0054)	0.249 (0.0056)	0.906 (0.0203)	0.133 (0.003)	0.085 (0.0019)
	Two‐stage	**0.195 (0.0044)**	**0.194 (0.0043)**	**0.809 (0.0181)**	**0.113 (0.0025)**	**0.067 (0.0015)**
MSE	Unadjusted	0.071 (0.0032)	0.058 (0.0025)	0.824 (0.0359)	0.020 (0.0009)	0.009 (0.0004)
	Pooled	0.059 (0.0025)	0.062 (0.0028)	0.822 (0.0360)	0.018 (0.0008)	0.007 (0.0003)
	Two‐stage	**0.038 (0.0016)**	**0.038 (0.0017)**	**0.655 (0.0307)**	**0.013 (0.0006)**	**0.005 (0.0002)**
Coverage	Unadjusted		0.772 (0.0133)		0.863 (0.0109)	0.826 (0.017)
	Pooled		**0.813 (0.0123)**		**0.916 (0.0088)**	0.970 (0.0076)
	Two‐stage		0.761 (0.0135)		**0.915 (0.0088)**	**0.962 (0.0086)**
Rel. error	Unadjusted		−**5.16% (2.97)**		**2.81% (2.44)**	17.9% (3.81)
	Pooled		−28.3% (2.07)		−10.4% (2.13)	**5.30% (3.40)**
	Two‐stage		−25.3% (2.22)		−4.75% (2.25)	**7.20% (3.44)**

*Note*: Monte Carlo standard errors are shown in parentheses; best values (including all indistinguishable values under Monte Carlo error) for each criterion for each setting/number of studies are highlighted in bold font.

As can be seen in Tables 2 and 3, the performance of the bootstrap‐based uncertainty quantification was disappointing for small numbers of studies (m<30). When simulations contained m=3 large studies, the bootstrap procedure underestimated estimators' standard errors and produced confidence intervals with less than desired coverage. These results reflect that it is difficult to estimate between‐study variation when only a small number of studies are available. In these cases, the relative error was large and negative. When simulations instead contained ten or thirty large studies, we see that our proposed stratified bootstrap procedure has better performance with relative error closer to zero and the coverage of the percentile‐based 95% CIs for the pooled and two‐stage estimators much closer to nominal levels. Surprisingly, the bootstrap‐based results reflect a similar pattern in settings with smaller sample sizes but with generally improved coverage accompanied by occasionally higher relative error, as shown in Table 3.

**TABLE 3:** Coverage of the 95% percentile intervals and the relative error in the bootstrapped standard error estimates derived using the proposed stratified bootstrap procedure for simulations under Setting 2 with small study sizes; simulations with m=3 and m=10 studies had 1000 replications; simulations with m=30 studies had 500 replications; bootstrapped estimates were derived using 1000 bootstrap samples.

Metric	Estimator	m=3	m=10	m=30
Coverage	Unadjusted	0.907 (0.0092)	0.959 (0.0063)	0.902 (0.0133)
	Pooled	0.914 (0.0089)	0.970 (0.0054)	0.980 (0.0063)
	Two‐stage	0.927 (0.0082)	0.970 (0.0054)	0.980 (0.0063)
Rel. Error	Unadjusted	10.4% (3.15)	27.7% (3.00)	28.6% (4.14)
	Pooled	−3.66% (2.51)	14.7% (2.68)	16.3% (3.76)
	Two‐stage	−0.91% (2.49)	13.0% (2.65)	22.1% (3.95)

*Note*: Monte Carlo standard errors are shown in parentheses.

## 6 Real Data Examples

In this section, we present two real data applications. Section 6.1 considers an application to HALT‐C, a multi‐center clinical trial where we expect few differences across the collection of studies. Section 6.2 considers an application to a collection of RCTs studying a therapy treatment for pediatric TBI that demonstrates the very different situation where we observe many differences across the collection of studies.

### 6.1 Application to HALT‐C

The HALT‐C [29] study enrolled patients at ten research centers in the United States who were 18 years of age or older, tested positive for Hepatitis C, had significant liver scarring, and failed to respond to standard treatment. At each center, patients were randomly assigned to treatment with peginterferon alfa‐2a or a control. For our analysis, we consider each center to be a separate study, so that m=10. We identified a target population more relevant to the general United States population using data from the National Health and Nutrition Examination Survey (NHANES), as described below. Our goal is to use the HALT‐C data to estimate the TATE of peginterferon alfa‐2a on platelet count change from baseline to nine months of treatment, where platelets were quantified as number ×1000/${\text{mm}}^{3}$. This goal is similar to those of analyses conducted by Dahabreh et al. [12, 13].

We aimed to construct a target population with exclusion criteria similar to those used in HALT‐C. NHANES is a complex survey run in two‐year cycles that utilizes personal interviews and physical examinations to collect demographic, health, and nutrition information at the person‐level [30]. To obtain a sizeable target sample for this exercise, we considered participants in the following NHANES cycles: 2007–2008, 2009–2010, 2011–2012, 2013–2014, 2015–2016, and 2017–2018 [30, 31, 32]. We included only those who were 18 years of age or older and tested positive for Hepatitis C. Because NHANES does not contain measures of the other HALT‐C eligibility requirements, we somewhat unrealistically assumed that these individuals also had significant liver scarring and failed to respond to standard treatment.

**FIGURE 1:**
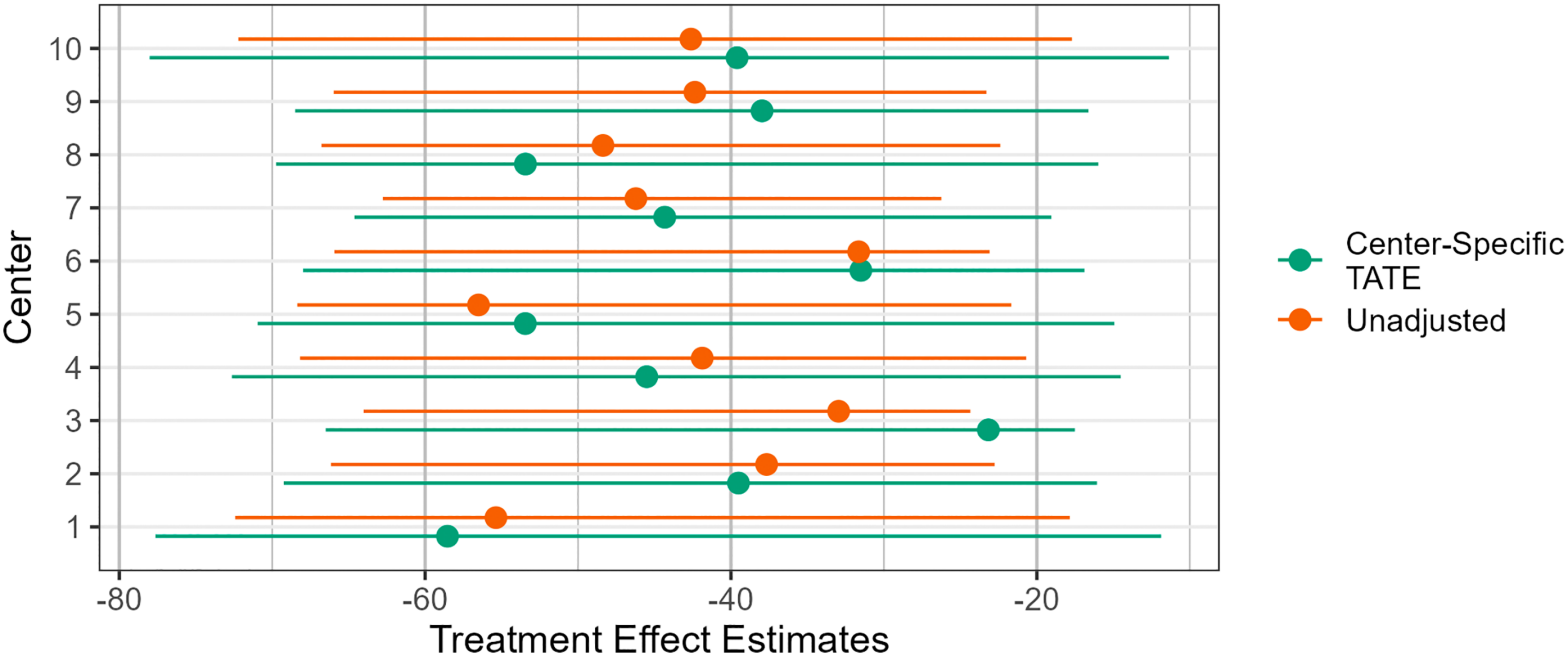
Unadjusted treatment effect estimates and center‐specific TATE estimates from each of the ten centers included in HALT‐C; plotted intervals are 95% bootstrapped percentile intervals.

We utilized the following baseline covariates: Age, sex, body mass index (BMI), and hemoglobin levels in g/dL. For ease, we restricted our target population to those with complete baseline covariate data and used only study observations with complete outcome and baseline covariate data. This resulted in an overall sample size of n=1342, with 348 observations in the target population sample—sizes similar to our small study size simulations. Baseline covariate summaries by study, including baseline platelet count, can be found in Table 4. Naïve center‐specific unadjusted treatment effect estimates along with bootstrap‐based 95% CIs are shown in Figure 1.

**TABLE 4:** HALT‐C baseline covariate summaries; categorical summaries shown as count (%) and continuous summaries shown as mean (SD).

	Target	Center 1	Center 2	Center 3	Center 4	Center 5
	(n=348)	(n=49)	(n=100)	(n=139)	(n=70)	(n=78)
Platelet count^a^	203 (62.8)	175 (77.1)	162 (67.2)	167 (68.1)	166 (57.3)	179 (64.2)
Age^b^	54.0 (10.0)	50.6 (6.91)	49.8 (6.53)	50.9 (6.34)	50.5 (8.31)	49.2 (6.47)
Female	111 (31.9%)	11 (22.4%)	29 (29.0%)	33 (23.7%)	23 (32.9%)	24 (30.8%)
BMI^c^	28.3 (7.02)	30.1 (6.14)	29.8 (6.13)	29.2 (4.65)	30.1 (6.10)	30.1 (5.34)
Hemoglobin^d^	14.5 (1.56)	15.0 (1.35)	14.8 (1.18)	15.7 (1.44)	14.9 (1.40)	14.9 (1.38)

	Center 6	Center 7	Center 8	Center 9	Center 10
	(n=110)	(n=205)	(n=94)	(n=100)	(n=49)
Platelet Count^a^	156 (61.8)	173 (68.4)	157 (68.1)	153 (52.8)	162 (71.4)
Age^b^	52.2 (8.37)	49.7 (7.46)	49.2 (6.71)	50.1 (7.38)	50.2 (6.90)
Female	34 (30.9%)	61 (29.8%)	27 (28.7%)	32 (32.0%)	12 (24.5%)
BMI^c^	29.8 (5.76)	29.9 (5.29)	30.5 (5.11)	29.9 (5.55)	30.4 (5.30)
Hemoglobin^d^	14.7 (1.55)	15.0 (1.42)	14.9 (1.56)	14.9 (1.28)	14.9 (1.40)

^a^ In $\times 1000/{\text{mm}}^{3}$.

^b^ In years.

^c^ In kg/$m^{2}$.

^d^ In g/dL.

Because the unadjusted estimates are simply the difference between the average outcome among the treated and the average outcome among the control within a center's sample, they are not directly causally interpretable for our target population. To demonstrate the first step in our two‐step procedure, Figure 1 also includes center‐specific TATE estimates that incorporate the target population baseline covariate distribution via an existing method for extending inferences from a single study. As expected, these causally interpretable estimates differ from the unadjusted ones. After adjusting each center's estimate to respect the target population, we see that the resulting estimates are fairly homogeneous, suggesting that there may be minimal heterogeneity in conditional treatment effects across centers. This is not particularly surprising because we are considering a collection of centers from the same larger RCT, which implies relative homogeneity of treatment application, supportive care. We also note that the adjusted estimate CI widths are larger, reflecting the effective reduction in sample size due to the individual weights.

We implemented our two‐stage TATE estimator similarly to our simulation studies, estimating the propensity of treatment for each individual using a logistic regression model and center‐specific estimates of the odds of participation in each study via a multinomial logistic regression model. All models included the main effects of age, sex, BMI, and hemoglobin level. Variance estimates and confidence intervals were obtained using the stratified bootstrap procedure with 10 000 replications. We found that treatment with peginterferon alfa‐2a has a TATE on 9‐month change in platelet count of −42.74 with a 95% CI of (−51.80, −34.15).

We expect the performance of the two‐stage estimator to depend on the data's alignment with Assumptions ℬ. In this example, we believe that most of these assumptions are reasonable. Because a randomized trial was conducted at each center, it is reasonable to expect that within each study, we have consistency of potential outcomes, exchangeability of potential outcomes, and positivity of treatment assignment within the study, which are, respectively, Assumptions B1, A2, and A3. Assumption B4 also likely follows from the multi‐center RCT setting. Because the trial conduct across centers is relatively homogeneous (e.g., same inclusion criteria, protocol, and procedures), we may expect that an individual's ATE, conditional on their covariates, does not depend on the center at which they were enrolled. If we further expect that the observations in the target population will have similar conditional ATEs to the study observations, it is reasonable to assume that Assumption B4 holds.

The final identifiability assumption, Assumption B5, indicates the positivity of participation in any study for all members of the target population, conditional on baseline covariates. We empirically explored the plausibility of this assumption by estimating the probability that each member of the target population would participate in the trial at each center via a collection of logistic regressions. As summarized in Table 5, we estimate that on average, observations in the target sample have a substantially positive probability of participation in each of the studies. Though the lower end of the ranges of predicted values is quite small, we believe that Assumption B5 largely holds for this example.

**TABLE 5:** Summaries of predicted probabilities of each member of the target population sample participating in the studies considered in the HALT‐C example.

Center	Mean	Std. deviation	Range
1	0.120	0.059	(0.027, 0.402)
2	0.214	0.092	(0.058, 0.505)
3	0.247	0.150	(0.015, 0.828)
4	0.162	0.074	(0.037, 0.488)
5	0.174	0.096	(0.031, 0.516)
6	0.236	0.058	(0.128, 0.465)
7	0.342	0.136	(0.076, 0.747)
8	0.199	0.107	(0.041, 0.58)
9	0.214	0.089	(0.055, 0.523)
10	0.120	0.065	(0.027, 0.393)

It is important to note that, while this analysis was an interesting example, these results should not be interpreted clinically without further scientific consideration of appropriate baseline covariates and model specification. We are also likely violating a core assumption of positivity of study participation because we were not able to obtain information about liver scarring and previous treatment from the NHANES data. Finally, because we utilized data from multiple cycles of a complex survey to construct our target population without incorporating survey weights into our analyses, the target population we are extending to is a contrived population from which our subset of NHANES participants could be an SRS, and results should certainly not be considered to apply to all residents of the United States who are over 18 years of age and Hepatitis C positive.

### 6.2 Application to TBI

A collection of four RCTs that studied the effect of online family problem‐solving therapy (OFPST) on symptoms in children who had been hospitalized for moderate‐to‐severe TBI provides an opportunity to explore a more heterogeneous collection of datasets [33, 34, 35, 36]. Each study randomly assigned children, along with their caregivers, to treatment with OFPST plus access to internet resources or to control, which was access to internet resources only or usual care. Enrollment criteria for each study were based on the child's age, in years, and their time since injury, in months. More information about each study and its recruitment criteria can be found in Table 6. Our goal is to estimate the TATE of six months of OFPST on the change in the Child Behavior Checklist's (CBCL) externalizing problems score from baseline to six months.

**TABLE 6:** Trial and recruitment summaries for studies included in our application to studies on pediatric TBI.

Study	Clinical trial number	Control	Age^a^	Time since injury^b^
CDC [37]	NCT00178022	Usual care	5–18	0–24
Online R21 [33]	—	Internet resource comparison	5–18	0–24
Original TOPS [34]	NCT00409058	Internet resource comparison	11–18	0–24
TOPS‐RRTC [35]	NCT01042899	Internet resource comparison	11–18	0–18

*Note*: —, study predated clinicaltrials.gov.

^a^ In years.

^b^ In months.

Our target population consists of all children aged 11 to 13 years who have experienced a moderate‐to‐severe TBI within the last three months. Covariate data for this population comes from a subset of a cohort study on children between the ages of 6 and 13 who had experienced a moderate‐to‐severe TBI [38]. We consider the subset of the cohort participants who were aged 11 to 13 years and had experienced a moderate‐to‐severe TBI within the last three months to be a simple random sample from the target population. We chose this subset to improve the plausibility of the positivity assumption A5, as discussed below.

CBCL externalizing problems scores were examined as t‐scores on the Externalizing Problems scale, where smaller scores indicate more well‐adjusted externalizing behaviors. Therefore, a negative treatment effect would indicate that OFPST is beneficial. We considered the following baseline covariates: Age in years, time since injury in months, sex, and severity of TBI. For ease, we only considered target observations with complete baseline covariate data and study observations with complete outcome and baseline covariate data. Our overall sample size was n=241 with 55 observations in the target population sample. Baseline covariate summaries by study, including baseline externalizing problems score, can be found in Table 7.

**TABLE 7:** TBI baseline covariate summaries; categorical summaries shown as Count (%); continuous summaries shown as Mean (SD).

	Target	CDC	Online R21	Original TOPS	TOPS‐RRTC
	(n=55)	(n=39)	(n=34)	(n=35)	(n=72)
Externalizing problems score	51.6 (10.3)	55.1 (12.8)	55.0 (12.5)	53.4 (10.6)	51.9 (11.9)
Age^a^	12.0 (0.546)	12.0 (3.52)	11.3 (3.18)	14.3 (2.35)	14.8 (2.03)
Time since injury^a^	0.0603 (0.0314)	0.346 (0.270)	1.09 (0.601)	0.807 (0.423)	0.490 (0.347)
Male	41 (74.5%)	22 (56.4%)	19 (55.9%)	17 (48.6%)	51 (70.8%)
Severe TBI	15 (27.3%)	12 (30.8%)	11 (32.4%)	14 (40.0%)	29 (40.3%)

^a^ In years.

**FIGURE 2:**
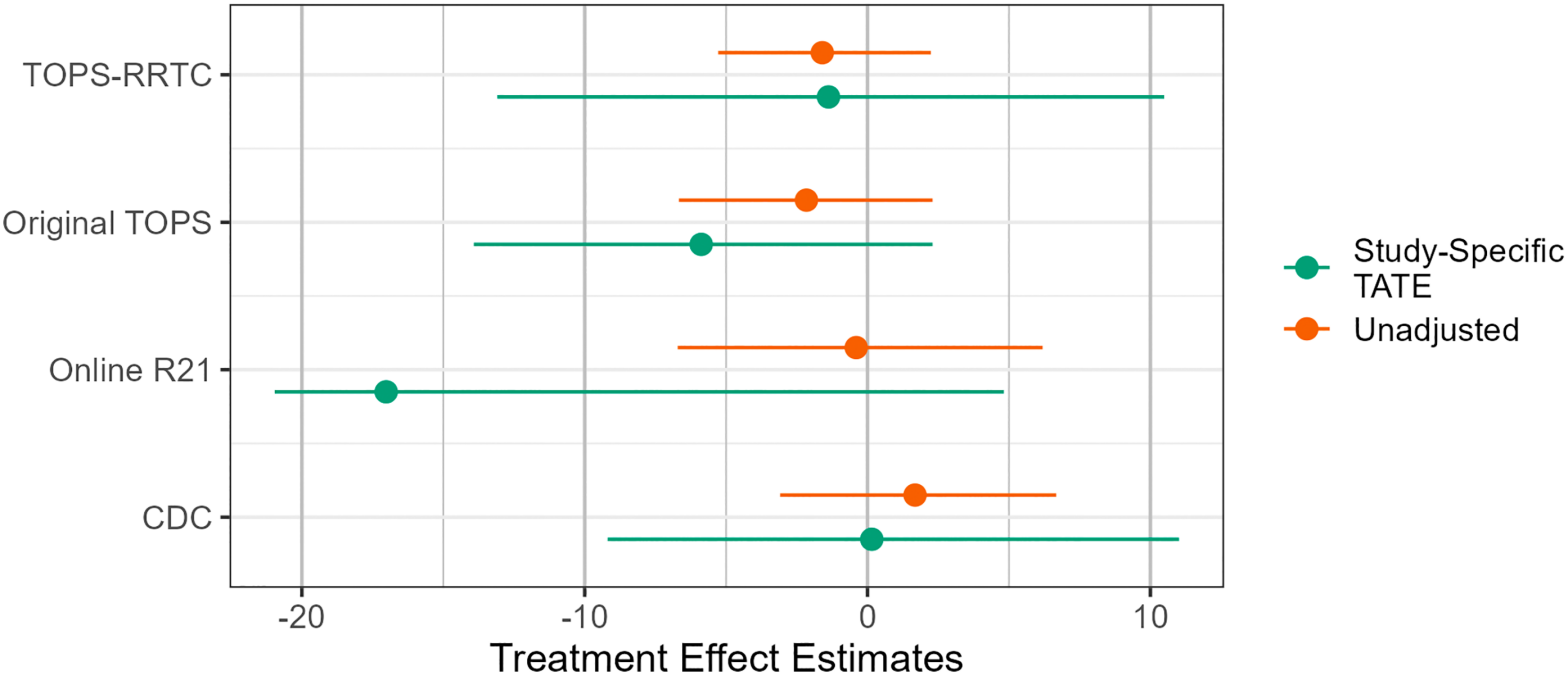
Unadjusted treatment effect estimates and study‐specific TATE estimates from each of the four pediatric TBI studies in our collection; plotted intervals are 95% bootstrapped percentile intervals.

Recall that each study considered in this analysis was a clinical trial considering a different population of interest. If we were to obtain an overall estimate by pooling these unadjusted estimates, as would typically be done in a traditional two‐stage meta‐analysis, it would not be clear what population the resulting estimate relates to. Figure 2 was constructed with the same methodology as Figure 1. It shows that in this case, causally‐interpretable study‐specific TATE estimates may be more heterogeneous than their naïve unadjusted counterparts, which are relatively homogeneous. This suggests that there may be across‐study heterogeneity in treatment effects.

We again estimated the TATE using logistic and multinomial models. All models included main effects of age and sex, an indicator of having experienced a severe TBI, and a quadratic term for age. We used 10 000 bootstrap replicates to estimate the variances and CIs. We estimated that the TATE of six months of OFPST on externalizing problems in children aged 11 to 13 years who had experienced a moderate‐to‐severe TBI within the last three months is −4.52 with a 95% CI of (−11.98, 2.78). Given the small size of our dataset, it is not surprising that we did not obtain a statistically significant effect estimate, as is possible within each original randomized trial analyzed according to the original study design. Additionally, it is important to note that these results should not be interpreted clinically without further scientific consideration of appropriate baseline covariates and model specification.

We note that our bootstrap procedure produced 55 missing values due to issues with positivity of study membership. This is a rate of 0.55%. To evaluate the sensitivity of our results to these missing values, we considered the percentile intervals that would result if all of the replications with missing values had estimates that were lower than all other estimates, the same as the mean estimate, or higher than all other estimates. The resulting intervals are shown in Table 8. If the TATE estimates from the bootstrap replications that produced missing values were near the mean estimate for a given estimator, we would see minimal changes in the percentile intervals. Even if the missing TATE estimates were more extreme, all lower or higher than the other bootstrapped estimates, we do not see large differences in the percentile intervals, and we observe no changes in statistical significance.

**TABLE 8:** Percentile intervals from the TBI analyses calculated considering missing values as lower than all other estimates, to be the same as the mean estimate, and as higher than all other estimates.

Supposed location of missing values	95% percentile interval
Lower than all estimates	(−12.48, 2.78)
At the mean estimate	(−11.97, 2.78)
Higher than all estimates	(−11.97, 3.17)

Again, it is important to consider the plausibility of our Assumption ℬ when evaluating the two‐stage estimator results. As in the HALT‐C example, the RCT study design of the individual studies leads to our concluding that Assumptions B1, A2, and A3 likely hold. (Because the studies in our collection were not part of one larger trial, Assumption A1 is less likely to hold.) Assumption B4, which allows for between‐study heterogeneity in conditional ATEs, may still be reasonable so long as we are willing to assume that the conditional ATE in the target population is the expected value of the conditional ATEs in the studies. This is a strong assumption, which we discuss further in Section 7.

With careful consideration of the range of feasible target populations, the positivity of participation in any study for each observation in the target population (Assumption A5) may also be reasonable. Age and time since injury (TSI) are two variables that may be important for estimating TATE. Table 9 contains summaries of these variables by study. It shows that the target sample (restricted to ages 11–13 by design) has reasonably good overlap with respect to age for all four studies, but its range of TSI has minimal overlap with that for the Online R21 study sample and no overlap with the Original TOPS study sample. This lack of overlap creates practical positivity issues if we were to also include TSI among the set of covariates X in Assumption A5. Thus, for this example, we excluded time since injury from all analyses, and therefore assume that its exclusion does not violate the other identifiability assumptions. Because TSI is likely a practically important variable in TBI treatment, we are concerned about this choice. This example demonstrates the trade‐off that our two‐stage estimator makes, allowing treatment effect heterogeneity but instead requiring a more restrictive set of assumptions for covariate overlap. Nonetheless, empirical checks based on models fit without a main effect for TSI showed predicted probabilities of participation far enough from zero to avoid concern about practical positivity violations. Summaries of the predicted probabilities can be found in Table 10.

**TABLE 9:** Summaries of eligibility criteria for the TBI studies and the sample from the target population are shown as mean [min, max].

	Target	CDC	Online R21	Original TOPS	TOPS‐RRTC
	(n=55)	(n=45)	(n=34)	(n=35)	(n=72)
Age^a^	12.0 [11.0, 13.0]	12.0 [5.31, 17.4]	11.3 [6.47, 18.0]	14.3 [11.1, 18.4]	14.8 [11.3, 18.5]
TSI^a^	0.0603 [0.0177, 0.156]	0.359 [0.0600, 0.950]	1.09 [0.150, 2.26]	0.807 [0.240, 1.68]	0.490 [0.0600, 1.51]

Abbreviation: TSI, time since injury.

^a^ In years.

**TABLE 10:** Summaries of predicted probabilities of each member of the target population sample participating in the studies considered in the application to studies on pediatric TBI.

Study	Mean	Std. deviation	Range
CDC	0.150	0.104	(0.043, 0.467)
Online R21	0.094	0.117	(0.020, 0.570)
Original TOPS	0.196	0.149	(0.076, 0.727)
TOPS‐RRTC	0.249	0.134	(0.142, 0.659)

## 7 Discussion

In this article, we proposed a class of estimators for extending inferences from a collection of RCTs that allows for between‐study heterogeneity in conditional ATEs given baseline covariates. Our new class of estimators enables us to extend inferences from a collection of RCTs in some settings where we expect differences in the effect of treatment across the studies included in our analyses, even after controlling for measured baseline covariates. The long history of random‐effect method development in traditional meta‐analysis and meta‐regression suggests that such heterogeneity is expected in practice, and that study membership may be a surrogate variable that captures a collection of unmeasured differences across the collection of studies. Our method is inspired by and effectively combines these traditional methods for two‐stage meta‐analysis with methods for extension from a single study. We proposed a new collection of sufficient identifiability assumptions for our heterogeneous setting that adapts assumptions previously proposed for the pooled approach by Dahabreh et al. [12, 13]. We also showed that, under these new assumptions, the TATE is identifiable and outlined a conjecture that our proposed two‐stage estimator with even study‐level weights is a consistent estimator of the TATE with the addition of a collection of regularity conditions. This consistency would hold when the number of studies grows with the number of observations at a bounded rate and the treatment and study membership models are correctly specified. Finally, we proposed a stratified bootstrap procedure to estimate variance and obtain CIs.

The TATE estimator performed well in the simulation settings we considered. They showed negligible bias and low empirical standard error as compared to the existing pooled estimator. Unfortunately, our simulations also demonstrated that our proposed bootstrap‐based uncertainty estimation performs poorly when there are only a small number of studies (i.e., m is small)—the norm in meta‐analysis. We illustrated the implementation of the two‐stage estimator in two very different realistic applications based on real‐world data. The HALT‐C example demonstrated the successful use of the two‐stage estimator in the case where the studies are relatively homogeneous. This application had a similar sample structure to the simulation with m=10 and small study sample sizes, where we saw the estimator perform well. However, simulations suggest that our reported CI is less reliable. Practically speaking, this application also illustrates the potential difficulty of obtaining a meaningful sample from a particular target population of interest—an issue for all methods of extension.

Our TBI example involved much more heterogeneity across studies in general, and a setting where between‐study treatment effect heterogeneity is expected even after controlling for measured covariates. We saw the practical positivity issues that can arise from a lack of overlap between the covariate spaces for the studies and for the target population, even when the needed theoretical positivity assumptions are reasonable. We expect these kinds of issues will arise more frequently when applying our proposed two‐stage method than existing pooled methods because we require that each study can be separately used to generalize to the target population (rather than the pooled study samples). Because existing pooled approaches are not appropriate for settings with between‐study effect heterogeneity, practical positivity issues may leave us with a choice among a collection of inappropriate methods in such settings. We hope that future extensions of our method may overcome this limitation.

To allow for between‐study heterogeneity in conditional treatment effects across studies, we relaxed Assumption A4 to assume that for each set of baseline covariates possible in the target population, the study‐specific conditional ATEs follow some distribution with an expected value that equals the conditional ATE in the target population. This choice was inspired by traditional meta‐analytic approaches, and can be thought of as reflecting the “average” of the TATEs that one might observe under the distribution of various study situations. Of course, this reasoning returns us to the problem that study settings are generally not SRSs from a meaningful distribution of possible study designs. Nonetheless, we believe that our proposed assumption is more realistic than those proposed for existing methods, even as we acknowledge that this is still quite a strong assumption and may often be unreasonable. One set of future work that would make our proposed method more useful is to create guidelines for evaluating when utilizing a two‐stage approach to extending inference improves the performance of estimators enough to justify the additional complication. Another direction for future work would be to further relax this assumption to allow the target population average to be offset from the center. Such an offset could be driven by subject‐matter expertise or could be inspired by the substantial body of work related to missing data and non‐normal random effect distributions in traditional meta‐analysis. A third approach could be similar to that pursued by Clark et al. [18], where the target population random effect was assumed to be drawn from the same distribution as the studies.

In this article, we focused on methods for extending inferences that involve taking weighted averages of observed outcomes, which we refer to as weighted methods. These methods are appealing, in part, because they do not require us to assume that we can develop an appropriate model for the outcome, a task that could be challenging even for subject‐matter experts. Nonetheless, another approach to extending inferences would involve modeling the conditional expectation of the potential outcomes in the target population. Such approaches have been proposed for extending inferences from a single study (e.g., see Buchanan et al. [1], Dahabreh et al. [6, 12]). For extension from a collection of studies, approaches that rely on pooling the study data have also been proposed and, like the pooled weighted estimator we considered in this work, assume that an observation's expected treatment effect conditional on their covariates does not depend on what study they were enrolled in (e.g., see Dahabreh et al. [12, 13], Steingrimsson et al. [17]). We believe that the ideas used in this work could be used in concert with our two‐stage framework to develop a model‐based approach that allows for between‐study heterogeneity in observations' conditional expected treatment effects. Future work in these or similar directions could move our proposed class of estimators into a new phase or new cycle of methodological development [16] that would move causally‐interpretable meta‐analysis to the next level of accuracy and usefulness.

## Disclosure

The authors have nothing to report.

## Conflicts of Interest

The authors declare no conflicts of interest.

## Supporting information


**Data S1.** Supporting Information.

## Data Availability

The data that support the application to the Hepatitis C Antiviral Long‐Term Treatment Against Cirrhosis are available from NIDDK. Restrictions apply to the availability of these data, which were used under license for this study. Data are available from https://repository.niddk.nih.gov/studies/halt‐c/ with the permission of NIDDK. The data that support the application to a collection of studies on pediatric traumatic brain injury are available on reasonable request from the corresponding author. The data are not publicly available due to privacy or ethical restrictions.

## Appendix A Identifiability of the TATE

In this appendix, we prove the identifiability Theorem 1 introduced in Section 4.1.1. In addition to the data situation described in Section 2, let S∈{0,𝒮} and A∈𝒜 be random variables for study membership and treatment, respectively. Further, assume the collection of assumptions, denoted Assumption ℬ.

### Proof of Equation (6)

Let $\Delta \left(X,S\right)\equiv E_{Y^{a},Y^{a^{\prime }}}\left(Y^{a}-Y^{a^{\prime }}|X,S\right)$. Then we have:

Δ=EYa,Ya′Ya−Ya′|S=0=EXEYa,Ya′Ya−Ya′|X,S=0S=0 by iterated expectations=EXES[Δ(X,S)|S∈S]|S=0  by Assumptions B4 and A5=ESEX[Δ(X,S)|S=0]|S∈S by Fubini's Theorem and the regularity condition=ESΔS|S∈S

□

### Proof of Equation (2)

We show the proof for discrete X, but analogous arguments hold for X with other distributions as long as the joint distribution of X and S exists. For each s∈𝒮, we can write

(A1)
Δs=EXEYa,Ya′Ya−Ya′X,S=sS=0=EXEYaYaX,S=s,A=aS=0 −EXEYa′Ya′X,S=s,A=a′S=0 by Assumptions A2 and A3=EXEY(Y|X,S=s,A=a)|S=0 −EXEYY|X,S=s,A=a′S=0 by Assumption B1

Notice that, for all a∈𝒜 and s∈𝒮,

(A2)
EX,A,Y[w(a,s)Y|S=s]=EX,A,YI(A=a)P(A=a|X,S)P(S=0|X)P(S=s|X)YS=s=EXEA,YI(A=a)P(A=a|X,S=s)P(S=0|X)P(S=s|X)YX,S=sS=s by the definition of conditional expectation=EXEA,Y[I(A=a)Y|S=s,X]P(A=a|X,S=s)P(S=0|X)P(S=s|X)S=s

Additionally, we can write

(A3)
EA,Y[I(A=a)Y|X,S=s]=EAI(A=a)EY[Y|X,S=s,A]X,S=s bythedefinitionofconditionalexpectation=∑a*∈AIa*=aEYY|X,S=s,A=a*PA=a*|X,S=s because A isdiscrete=EY[Y|X,S=s,A=a]P(A=a|X,S=s)

because $I\left(a^{*}=a\right)=0$ for all $a^{*}\neq a$. Equations (A2) and (A3) together lead to

(A4)
$$\begin{array}{ll} E_{X,A,Y} & \left[w\left(a,s\right)Y|S=s\right]\\ = & \sum_{x}\frac{P\left(S=0|X=x\right)}{P\left(S=s|X=x\right)}E_{Y}\left[Y|X=x,S=s,A=a\right]P\left(X=x|S=s\right)\\ & \text{ assuming }X\text{ is discrete}\\ = & \;\frac{P\left(S=0\right)}{P\left(S=s\right)}\sum_{x}E_{Y}\left[Y|X=x,S=s,A=a\right]P\left(X=x|S=0\right)\\ & \text{by Bayes' Theorem}\\ = & \;\frac{P\left(S=0\right)}{P\left(S=s\right)}E_{X}\left\{\left.E_{Y}\left[Y|X,S=s,A=a\right]\right|S=0\right\} \end{array}$$

From Equations (A1) and (A4) we have that

(A5)
$$\begin{array}{c} \Delta_{s}=\frac{P\left(S=s\right)}{P\left(S=0\right)}E_{A,X,Y}\left[w\left(a,s\right)Y|S=s\right]-\frac{P\left(S=s\right)}{P\left(S=0\right)}E_{A,X,Y}\left[w\left(a^{\prime },s\right)Y|S=s\right] \end{array}$$

Next, notice that for all a∈𝒜 and s∈𝒮,

(A6)
EA,X(w(a,s)|S=s)=EA,XI(A=a)P(A=a|X,S=s)P(S=0|X)P(S=s|X)S=s=EXEAI(A=a)P(A=a|X,S=s)P(S=0|X)P(S=s|X)X,S=sS=s by the definition of conditional expectations=EX1P(A=a|X,S=s)EA(I(A=a)|X,S=s)P(S=0|X)P(S=s|X)S=s=EXP(S=0|X)P(S=s|X)S=s=∑xP(S=0|X=x)P(S=s|X=x)P(X=x|S=s)  assuming X is discrete=P(S=0)P(S=s)∑xP(X=x|S=0) by Bayes' Theorem=P(S=0)P(S=s)

Finally, by combining Equations (A5) and (A6) we have

$$\begin{array}{ll} \Delta_{s}= & \frac{E_{A,X,Y}\left(w\left(a,s\right)Y|S=s\right)}{E_{A,X}\left(w\left(a,s\right)|S=s\right)}-\frac{E_{A,X,Y}\left(w\left(a^{\prime },s\right)Y|S=s\right)}{E_{A,X}\left(w\left(a^{\prime },s\right)|S=s\right)}\\ = & \;E_{A,X,Y}\left(\left[\frac{w\left(a,s\right)}{E_{X,A}\left(w\left(a,s\right)|S=s\right)}-\frac{w\left(a^{\prime },s\right)}{E_{X,A}\left(w\left(a^{\prime },s\right)|S=s\right)}\right]Y|S=s\right) \end{array}$$

□
