# Supplementary material for: A Two‐Stage Method for Extending Inferences From a Collection of Trials
Source: Stat Med. 2025 Jun 5;44(13-14):e70146. doi: 10.1002/sim.70146 (PMC12138745; doi:10.1002/sim.70146)
Supplement: Supplementary file 1 — Data S1. Supporting Information. [file SIM-44-0-s001.zip › suppinfo.pdf]

Supporting Materials for "A Two-Stage Method for Extending Inferences from a Collection of Trials"

Nicole Schnitzler[1] and Eloise Kaizar[2]

[1] Ohio Colleges of Medicine Government Resource Center, The Ohio State University

[2] Department of Statistics, The Ohio State University

This document contains supporting materials for "A Two-Stage Method for Extending Inferences from a Collection of Trials". Section 1 contains details about our conjecture related to the consistency of our proposed two-stage estimator. Section 2 contains additional details about the paper's simulation studies, with full simulation results in Section 2.4.

1 Consistency of the Two-Stage Weighted Estimator of the TATE

We conjecture that our proposed two-stage estimator of the TATE with even study-level weights is a consistent estimator under the identifiability assumptions we presented and some regularity conditions if the number of studies is growing with the sample size at a bounded rate and the treatment and study membership models are correctly specified. This appendix contains a detailed outline of our reasoning for logistic treatment models and multinomial logistic study membership models.

1.1 Notation and Assumptions

Suppose that we have access to data on n observations from a collection of m RCTs, $\mathcal{S} = \{1, 2, ..., m\}$, and an SRS from a target population. Let Y_i, $i = 1, 2, ..., n$, be the outcome of interest, $\boldsymbol{X}_i$ be a length $q-1$ vector of baseline covariates, and $A_i \in \mathcal{A}$ be the treatment where $\mathcal{A}$ is a finite set. Let $S_i \in \{0, \mathcal{S}\}$ indicate study membership where $S_i = 0$ if observation i is in the target population. Assume the following regularity conditions:

(i) From each $s \in \mathcal{S}$ we have n_s observations $\{\boldsymbol{X}_i, S_i = s, A_i, Y_i\}$ that are independent and identically distributed for $i \in \{1, ..., n : S_i = s\}$ and $n = \sum_{s \in \mathcal{S}} n_s$.

(ii) From the target population we have n_0 observations $\{\boldsymbol{X}_i, S_i = 0\}$ that are independent and identically distributed for $i \in \{1, ..., n : S_i = 0\}$.

(iii) For each $s, k \in \mathcal{S}$ with $s \neq k$, $\{\boldsymbol{X}_i, S_i = s, A_i, Y_i\}$ is independent of $\{\boldsymbol{X}_j, S_j = k, A_j, Y_j\}$ for each $i \in \{1, ..., n : S_i = s\}$ and $j \in \{1, ..., n : S_j = k\}$.

(iv) For all $i, j \in \{1, 2, ..., n\}$ with $i \neq j$, $\{\boldsymbol{X}_i, S_i\}$ is independent of $\{\boldsymbol{X}_j, S_j\}$

(v) $E_S \left\{ E_X \left[\left| E_{Y^a, Y^{a'}} \left(Y^a - Y^{a'} | \boldsymbol{X}, S \right) \right| | S = 0 \right] | S \in \mathcal{S} \right\} < \infty$.

Additionally, assume that the collection of identifiability assumptions, Assumption $\mathcal{B}$,

B1. For all $s \in \mathcal{S}$, $a \in \mathcal{A}$, and $i \in \{1, 2, ..., n : S_i = s\}$, if $A_i = a$ then $Y_i^a = Y_i$.

A2. For all $s \in \mathcal{S}$, for all $a \in \mathcal{A}$, and for $\boldsymbol{x}$ such that $f(\boldsymbol{x}, S = s) > 0$,

$$E(Y^a | \boldsymbol{X} = \boldsymbol{x}, S = s, A = a) = E(Y^a | \boldsymbol{X} = \boldsymbol{x}, S = s).$$

A3. For all $s \in \mathcal{S}$, for all $a \in \mathcal{A}$, and for $\boldsymbol{x}$ such that $f(\boldsymbol{x}, S = s) > 0$,

$$P(A = a | \boldsymbol{X} = \boldsymbol{x}, S = s) > 0.$$

B4. For all $a, a' \in \mathcal{A}$ and for $\boldsymbol{x}$ such that $f(\boldsymbol{x}, S = 0) > 0$,

$$E(Y^a - Y^{a'} | \boldsymbol{X} = \boldsymbol{x}, S = 0) = E_S \left[E(Y^a - Y^{a'} | \boldsymbol{X} = \boldsymbol{x}, S) \middle| S \in \mathcal{S} \right].$$

A5. For all $s \in \mathcal{S}$ and for $\boldsymbol{x}$ such that $f(\boldsymbol{x}, S = 0) > 0$,

$$P(S = s | \boldsymbol{X} = \boldsymbol{x}) > 0.$$

hold where $f(\cdot)$ is used to denote densities.

With even study-level weights, our proposed two-stage estimator takes the form

$$\hat{\Delta}_{two} = \frac{1}{m} \sum_{s \in \mathcal{S}} \hat{\Delta}_s$$

where

$$\hat{\Delta}_s = \frac{1}{\sum_{i:S_i=s} \hat{w}_i(a, s)} \sum_{i:S_i=s} \hat{w}_i(a, s) Y_i - \frac{1}{\sum_{i:S_i=s} \hat{w}_i(a', s)} \sum_{i:S_i=s} \hat{w}_i(a', s) Y_i,$$

for each $s \in \mathcal{S}$. For each $a \in \mathcal{A}$ and $s \in \mathcal{S}$,

$$\hat{w}_i(a, s) = \frac{I(A_i = a)}{\hat{e}_a(\boldsymbol{X}_i, s)} \frac{\hat{p}(\boldsymbol{X}_i, 0)}{\hat{p}(\boldsymbol{X}_i, s)}$$

where

$$e_a(\boldsymbol{X}_i, s) = P(A = a|\boldsymbol{X}_i, S = s)$$
$$p(\boldsymbol{X}_i, s) = P(S = s|\boldsymbol{X}_i)$$
$$p(\boldsymbol{X}_i, 0) = P(S = 0|\boldsymbol{X}_i)$$

and corresponding quantities with hats are estimates of these probabilities.

For each $s \in \mathcal{S}$, we assume that for each $i \in \{1, 2, ..., n : S_i = s\}$, there exists a $\boldsymbol{\theta}_{0,s} \in \mathbb{R}^q$ such that

$$\begin{aligned} I(A_i = a) &\overset{indp}{\sim} \text{Bernoulli}(e_a(\boldsymbol{X}_i, s)) \\ e_a(\boldsymbol{X}_i, s) &= P(A_i = a|\boldsymbol{X}_i, S_i = s) = \text{expit}\left(\boldsymbol{V}_i^t \boldsymbol{\theta}_{0,s}\right) \end{aligned} \tag{1}$$

where $\boldsymbol{V}_i = (1, \boldsymbol{X}_i^t)^t \in \mathbb{R}^q$. Because the number of treatment models we fit depends on the number of studies and we would like to allow the number of studies to grow with our sample size, we reframe this collection of treatment models as one larger model with $q \times m$ parameters. If we define

$$\boldsymbol{\theta}_0 = (\boldsymbol{\theta}_{0,1}^t, ..., \boldsymbol{\theta}_{0,m}^t)^t \in \mathbb{R}^{qm}$$
$$\boldsymbol{Z}_{is} = I(S_i = s)\boldsymbol{V}_i \in \mathbb{R}^q$$
$$\boldsymbol{Z}_i = (\boldsymbol{Z}_{i1}^t, ..., \boldsymbol{Z}_{im}^t)^t \in \mathbb{R}^{qm}.$$

then we can write

$$e_a(\boldsymbol{X}_i, s) = P(A_i = a|\boldsymbol{X}_i, S_i = s) = \frac{\exp\{\boldsymbol{Z}_i^t \boldsymbol{\theta}_0\}}{1 + \exp\{\boldsymbol{Z}_i^t \boldsymbol{\theta}_0\}}$$
$$e_{a'}(\boldsymbol{X}_i, s) = P(A_i = a'|\boldsymbol{X}_i, S_i = s) = \frac{1}{1 + \exp\{\boldsymbol{Z}_i^t \boldsymbol{\theta}_0\}}$$

For each $i = 1, 2, .., n$, we also assume that there exists a $\boldsymbol{\beta}_{0,s} \in \mathbb{R}^q$ such that

$$\begin{aligned} (I(S_i = 0), ..., I(S_i = m)) &\overset{indp}{\sim} \text{MN}(1, p(\boldsymbol{X}_i, 0), ..., p(\boldsymbol{X}_i, m)) \\ \log\left(\frac{p(\boldsymbol{X}_i, s)}{p(\boldsymbol{X}_i, 0)}\right) &= \boldsymbol{V}_i^t \boldsymbol{\beta}_{0,s} \end{aligned} \tag{2}$$

for $s \in \mathcal{S}$ such that $\sum_{s \in \mathcal{S}} p(\boldsymbol{X}_i, s) = 1$ for all i. (Notice that, for ease, we are assuming that the same vector of coefficients yields the true treatment and study membership models.) If we define

$$\boldsymbol{\beta}_0 = (\boldsymbol{\beta}_{0,1}^t, ..., \boldsymbol{\beta}_{0,m}^t)^t \in \mathbb{R}^{mq}$$
$$\boldsymbol{U}_{is} = e_s \otimes \boldsymbol{V}_i \in \mathbb{R}^{mq}$$

where e_s is the sth column of the $m \times m$ identity matrix and $\otimes$ denotes the Kronecker product. Then $\boldsymbol{V}_i^t \boldsymbol{\beta}_{0,s} = \boldsymbol{U}_{is}^t \boldsymbol{\beta}_0$ and

$$p(\boldsymbol{X}_i, s) = P(S_i = s|\boldsymbol{X}_i) = \frac{\exp\{\boldsymbol{U}_{is}^t \boldsymbol{\beta}_0\}}{1 + \sum_{k=1}^m \exp\{\boldsymbol{U}_{ik}^t \boldsymbol{\beta}_0\}}$$
$$p(\boldsymbol{X}_i, 0) = P(S_i = 0|\boldsymbol{X}_i) = \frac{1}{1 + \sum_{k=1}^m \exp\{\boldsymbol{U}_{ik}^t \boldsymbol{\beta}_0\}}.$$

for $s \in \mathcal{S}$.

1.2 Consistency Conjecture

We conjecture that our proposed estimator $\hat{\Delta}_{two}$ with even study-level weights is a consistent estimator of the TATE

$$\Delta = E_{Y^a,Y^{a'}}(Y^a - Y^{a'}|S=0)$$

under the setting described in Section 1.1 as well as a collection of regularity conditions if the number of studies grows with the sample size at a bounded rate and the sizes of the target sample and each study sample are also growing with the overall sample size. In notation, this means that we conjecture that

$$\hat{\Delta}_{two} \xrightarrow{\mathbb{P}} \Delta \tag{3}$$

as $m \to \infty$ and $\min\{\tilde{\boldsymbol{n}}\} \to \infty$ where $\tilde{\boldsymbol{n}} = \{n_s\}_{s=0}^{m}$ if $m = o(g(n))$ for a carefully chosen function $g(\cdot)$ where $n = \sum_{s=0}^{m} n_s$.

1.3 Reasoning

From Appendix A, we know that under Assumption $\mathcal{B}$ and regularity condition (v)

$$\Delta = E_S(\Delta_S|S \in \mathcal{S}) = E_S(\Delta_S|S \neq 0) \tag{4}$$

where, for each $s \in \mathcal{S}$,

$$\Delta_s = E_{A,X,Y}\left(\left[\frac{w(a,s)}{E_{A,X}(w(a,s)|S=s)} - \frac{w(a',s)}{E_{A,X}(w(a',s)|S=s)}\right] Y \middle| S=s\right)$$

and, for each $s \in \mathcal{S}$ and $a \in \mathcal{A}$,

$$w(a,s) = \frac{I(A=a)}{P(A=a|\boldsymbol{X}, S=s)} \frac{P(S=0|\boldsymbol{X})}{P(S=s|\boldsymbol{X})}.$$

By Equation 4, if we can show that

$$\hat{\Delta}_{two} = \frac{1}{m}\sum_{s=1}^{m} \hat{\Delta}_s \xrightarrow{\mathbb{P}} E_S(\Delta_S|S \neq 0) \tag{5}$$

we will have that our two-stage estimator of the TATE with even study-level weights is consistent.

Next, by the weak law of large numbers, we have that

$$\frac{1}{m}\sum_{s=1}^{m} \Delta_s \xrightarrow{\mathbb{P}} E_S(\Delta_S|S \neq 0) \tag{6}$$

as $m \to \infty$ if the regularity condition

(vi) $\{\Delta_s\}_{s=1}^{m}$ are mutually independent with $E(\Delta_s) = E(\Delta_S|S \neq 0)$ for all $s \geq 1$ and $\frac{1}{m^2}\sum_{s=1}^{m} \text{Var}(\Delta_s) \to 0$ as $m \to \infty$

holds. Therefore, if we assume that (vi) holds and show that

$$\frac{1}{m}\sum_{s=1}^{m}\left(\hat{\Delta}_s - \Delta_s\right) \xrightarrow{\mathbb{P}} 0 \tag{7}$$

as $m \to \infty$ and $\min\{\tilde{\boldsymbol{n}}\} \to \infty$ with $m = o(g(n))$ for a carefully chosen function $g(\cdot)$, we will have that our two-stage estimator with even study-level weights is consistent because

$$\hat{\Delta}_{two} = \frac{1}{m}\sum_{s=1}^{m}\left(\hat{\Delta}_s - \Delta_s\right) + \frac{1}{m}\sum_{s=1}^{m}\Delta_s$$

For a fixed number of studies m, we can show the result in Equation 7 as $\min\{\tilde{\boldsymbol{n}}\} \to \infty$. First, notice that if the treatment model shown in Equation 1 and the study membership model shown in Equation 2 are correctly specified, $\hat{\boldsymbol{\theta}}_s$ and $\hat{\boldsymbol{\beta}}_s$ are the MLEs of $\boldsymbol{\theta}_{0,s}$ and $\boldsymbol{\beta}_{0,s}$, respectively, for each $s = 1, 2, ..., m$, and regularity conditions (i) to (iv) hold, we have that

$$\begin{aligned} \hat{e}_a(\boldsymbol{X}_i, s) &= \text{expit}\left(\boldsymbol{V}_i^t\hat{\boldsymbol{\theta}}_s\right) \xrightarrow{\mathbb{P}} e_a(\boldsymbol{X}_i, s) = \text{expit}\left(\boldsymbol{V}_i^t\boldsymbol{\theta}_{0,s}\right) \\ \hat{e}_{a'}(\boldsymbol{X}_i, s) &= 1 - \text{expit}\left(\boldsymbol{V}_i^t\hat{\boldsymbol{\theta}}_s\right) \xrightarrow{\mathbb{P}} e_{a'}(\boldsymbol{X}_i, s) = 1 - \text{expit}\left(\boldsymbol{V}_i^t\boldsymbol{\theta}_{0,s}\right) \end{aligned} \tag{8}$$

as $n_s \to \infty$ for all $s = 1, 2, ..., m$ and

$$\frac{\hat{p}(\boldsymbol{X}_i, s)}{\hat{p}(\boldsymbol{X}_i, 0)} = \exp\{\boldsymbol{V}_i^t\hat{\boldsymbol{\beta}}_s\} \xrightarrow{\mathbb{P}} \frac{p(\boldsymbol{X}_i, s)}{p(\boldsymbol{X}_i, s)} = \exp\{\boldsymbol{V}_i^t\boldsymbol{\beta}_{0,s}\} \tag{9}$$

as $\min\{\tilde{\boldsymbol{n}}\} \to \infty$ under some usual regularity conditions. Then, it can be shown that for all $s = 1, 2, ..., m$

$$\begin{aligned} \frac{1}{n_s}\sum_{i:S_i=s}\hat{w}_i(a, s) &\xrightarrow{\mathbb{P}} E_{A,X}\left(w(a, s)|S = s\right) \\ \frac{1}{n_s}\sum_{i:S_i=s}\hat{w}_i(a, s)Y_i &\xrightarrow{\mathbb{P}} E_{A,X,Y}\left(w(a, s)Y|S = s\right) \end{aligned} \tag{10}$$

if $\min\{\tilde{\boldsymbol{n}}\} \to \infty$. For details of this result, see Lemma 1 in Section 1.4. Then for each $s = 1, 2, ..., m$,

$$\begin{aligned} \hat{\Delta}_s =& \frac{\sum_{i:S_i=s}\hat{w}_i(a, s)Y_i}{\sum_{i:S_i=s}\hat{w}_i(a, s)} - \frac{\sum_{i:S_i=s}\hat{w}_i(a', s)Y_i}{\sum_{i:S_i=s}\hat{w}_i(a', s)} \\ \xrightarrow{\mathbb{P}}& \frac{E_{A,X,Y}(w(a, s)Y|S = s)}{E_{A,X}\left(w(a, s)|S = s\right)} - \frac{E_{A,X,Y}(w(a', s)Y|S = s)}{E_{A,X}(w(a', s)|S = s)} \\ & \textit{by continuous mapping theorem} \\ & = E_{A,X,Y}\left(\left[\frac{w(a, s)}{E_{A,X}\left(w(a, s)|S = s\right)} - \frac{w(a', s)}{E_{A,X}(w(a', s)|S = s)}\right] Y \middle| S = s\right) \\ & = \Delta_s \end{aligned} \tag{11}$$

as $\min\{\tilde{\boldsymbol{n}}\} \to \infty$. So, we then have that for fixed m

$$\frac{1}{m}\sum_{s=1}^{m}\left(\hat{\Delta}_s - \Delta_s\right) \xrightarrow{\mathbb{P}} 0$$

as $\min\{\tilde{\boldsymbol{n}}\} \rightarrow \infty$ if our models are correctly specified and regularity conditions (i) to (iv) hold.

Because we are able to show the result in Equation 7 for fixed m, it seems reasonable to expect that it should also hold for $m \rightarrow \infty$ so long as m is growing at a rate that is appropriately bounded. We conjecture that the rate needed is similar to $m = o\left(\frac{n}{\log(n)}\right)$ based on an application of results in He and Shao [1] that shows

$$\begin{aligned} ||\hat{\boldsymbol{\theta}}_n - \boldsymbol{\theta}_0|| &\xrightarrow{\mathbb{P}} 0 \\ ||\hat{\boldsymbol{\beta}}_n - \boldsymbol{\beta}_0|| &\xrightarrow{\mathbb{P}} 0 \end{aligned} \tag{12}$$

as $n, m \rightarrow \infty$ if $m = o\left(\frac{n}{\log(n)}\right)$, regularity conditions Conditions (i)-(iv) hold, and the models specified in Equations 1 and Equation 2 are correct under the following additional regularity conditions:

(vii). $\sum_{i=1}^{n} ||\boldsymbol{V}_i||^2 = O(nm)$

(viii). $\sup_{\boldsymbol{\alpha},\boldsymbol{\gamma} \in H_{qm}} \sum_{i=1}^{n} (\boldsymbol{\alpha}^t \boldsymbol{Z}_i)^2 (\boldsymbol{\gamma}^t \boldsymbol{Z}_i)^2 = O(n)$

(ix). $\sup_{\boldsymbol{\alpha},\boldsymbol{\gamma} \in H_{qm}} \sum_{i=1}^{n} (\boldsymbol{\alpha}^t \boldsymbol{U}_{is})^2 (\boldsymbol{\gamma}^t \boldsymbol{U}_{is})^2 = O(n)$ for all $s \in \mathcal{S}$

(x). $\lim\inf_{n\rightarrow\infty} \lambda_{min}(\boldsymbol{D}_{n,s}) > 0$ for each $s \in \mathcal{S}$ where

$$\boldsymbol{D}_{n,s} = \frac{1}{n} \sum_{i=1}^{n} I(S_i = s) \frac{\exp\{\boldsymbol{V}_i^t \boldsymbol{\theta}_{0,s}\}}{1 + \exp\{\boldsymbol{V}_i^t \boldsymbol{\theta}_{0,s}\}} \boldsymbol{V}_i^t \boldsymbol{V}_i.$$

(xi). $\lim\inf_{n\rightarrow\infty} \lambda_{min}(\boldsymbol{G}_n) > 0$ where

$$\boldsymbol{G}_n = \frac{1}{n} \sum_{i=1}^{n} \sum_{s \in \mathcal{S}} \frac{\exp\{\boldsymbol{U}_{i,s}^t \boldsymbol{\beta}_0\}}{1 + \exp\{\boldsymbol{U}_{i,s}^t \boldsymbol{\beta}_0\}} \boldsymbol{U}_{i,s}^t \boldsymbol{U}_{i,s}.$$

where $||\boldsymbol{a}||$ is the L_2 norm of $\boldsymbol{a}$, $H_{qm} = \{\boldsymbol{\alpha} \in \mathbb{R}^{qm} : ||\boldsymbol{\alpha}|| = 1\}$, and λ_{min} denotes the smallest eigenvalue of a matrix. For a sketch of the application of the results from He and Shao [1] to our setting, see Lemma 2 in Section 1.4.

1.4 Supporting Results

Lemma 1: *For a fixed number of studies m,*

(i) $\frac{1}{n_s} \sum_{i:S_i=s} \hat{w}_i(a, s) \xrightarrow{\mathbb{P}} E_{A,X}\left(w(a, s)|S = s\right)$

(ii) $\frac{1}{n_s} \sum_{i:S_i=s} \hat{w}_i(a, s) Y_i \xrightarrow{\mathbb{P}} E_{A,X,Y}\left(w(a, s) Y|S = s\right)$.

for each $s = 1, 2, ..., m$ *and* $a \in \mathcal{A}$ *if* $\hat{e}_a(\boldsymbol{V}_i, s) \xrightarrow{\mathbb{P}} e_a(\boldsymbol{X}_i, s)$ *and* $\frac{\hat{p}(\boldsymbol{X}_i,s)}{\hat{p}(\boldsymbol{X}_i,s)} \xrightarrow{\mathbb{P}} \frac{p(\boldsymbol{X}_i,s)}{p(\boldsymbol{X}_i,s)}$ *as* $\min\{\tilde{\boldsymbol{n}}\} \rightarrow \infty$ *where* $\tilde{\boldsymbol{n}} = \{n_s\}_{s=0}^{m}$.

Proof of Lemma 1: For each $a \in \mathcal{A}$ and $s = 1, 2, ..., m$, define

$$\hat{\theta}_i(a, s) = \frac{e_a(\boldsymbol{X}_i, s)}{\hat{e}_a(\boldsymbol{X}_i, s)} \frac{\hat{p}(\boldsymbol{X}_i, 0)}{p(\boldsymbol{X}_i, 0)} \frac{p(\boldsymbol{X}_i, s)}{\hat{p}(\boldsymbol{X}_i, s)}.$$

Let $\hat{\theta}_{[j]}(a, s)$ is the jth largest $\hat{\theta}_i(a, s)$ for $i \in \{1, 2, ..., n : S_i = s\}$ and define

$$\begin{aligned} M_n(a, s) &= \max\left\{\left|C_{[1]}(a, s)\right|, \left|C_{[n_s]}(a, s)\right|\right\} \\ M_n^*(a, s) &= \max\left\{\left|C^*_{[1]}(a, s)\right|, \left|C^*_{[n_s]}(a, s)\right|\right\} \end{aligned}$$

for all $j = 1, 2, ..., n_s$, where

$$\begin{aligned} C_{[j]}(a, s) &= \frac{1}{n_s} \sum_{i:S_i = s} \hat{\theta}_{[j]}(a, s) w_i(a, s) - E\left(w(a, s)|S = s\right) \\ C^*_{[j]}(a, s) &= \frac{1}{n_s} \sum_{i:S_i = s} \hat{\theta}_{[j]}(a, s) w_i(a, s) Y_i - E\left(w(a, s) Y|S = s\right). \end{aligned}$$

For each $a \in \mathcal{A}$ and $s = 1, 2, ..., m$,

$$\begin{aligned} &\left|\frac{1}{n_s} \sum_{i:S_i = s} \hat{\theta}_i(a, s) w_i(a, s) - E_{A,X}\left(w(a, s)|S = s\right)\right| \leq M_n(a, s) \\ &\Rightarrow P\left(\left|\frac{1}{n_s} \sum_{i:S_i = s} \hat{\theta}_i(a, s) w_i(a, s) - E_{A,X}\left(w(a, s)|S = s\right)\right| > \epsilon\right) \leq P(M_n(a, s) > \epsilon) \end{aligned}$$

for all $\epsilon > 0$. So, by Sub-Lemma 2.3, we have that

$$P\left(\left|\frac{1}{n_s} \sum_{i:S_i = s} \hat{\theta}_i(a, s) w_i(a, s) - E_{A,X}\left(w(a, s)|S = s\right)\right| > \epsilon\right) \to 0$$

for all $\epsilon > 0$ as as $n \to \infty$ such that $n_s \to \infty$ for all $s = 1, 2, ..., m$. This implies that

$$\frac{1}{n_s} \sum_{i:S_i = s} \hat{\theta}_i(l, s) w_i(l, s) \xrightarrow{\mathbb{P}} E_{A,X}\left(w(a, s)|S = s\right)$$

so, by Sub-Lemma 2.1, we have

$$\frac{1}{n_s} \sum_{i:S_i = s} \hat{w}_i(l, s) \xrightarrow{\mathbb{P}} E_{A,X}\left(w(a, s)|S = s\right)$$

as $\min\{\tilde{\boldsymbol{n}}\} \to \infty$ which proves Part (i). The proof for Part (ii) follows similarly, utilizing $C^*_{[j]}(a, s)$ and M_n^*

□

Sub-Lemma 1.1 *For each for each $s = 1, 2, ..., m$ and $a \in \mathcal{A}$,*

(i) $\frac{1}{n_s} \sum_{i:S_i = s} \hat{w}_i(a, s) = \frac{1}{n_s} \sum_{i:S_i = s} \hat{\theta}_i(a, s) w_i(a, s)$

(ii) $\frac{1}{n_s} \sum_{i:S_i = s} \hat{w}_i(a, s) Y_i = \frac{1}{n_s} \sum_{i:S_i = s} \hat{\theta}_i(a, s) w_i(a, s) Y_i$

where

$$\hat{\theta}_i(a,s) = \frac{e_a(\boldsymbol{X}_i,s)}{\hat{e}_a(\boldsymbol{X}_i,s)}\frac{\hat{p}(\boldsymbol{X}_i,0)}{p(\boldsymbol{X}_i,0)}\frac{p(\boldsymbol{X}_i,s)}{\hat{p}(\boldsymbol{X}_i,s)}$$

<u>Proof of Sub-Lemma 1.1</u>: For each for each $s = 1,2,...,m$, $a \in \mathcal{A}$, and $i = 1,2,..,n$, we can write

$$\begin{aligned}\hat{w}_i(a,s) &= \left[\frac{I(A_i=a)}{e_a(\boldsymbol{X}_i,s)}\frac{e_a(\boldsymbol{X}_i,s)}{\hat{e}_a(\boldsymbol{X}_i,s)}\right]\left[\frac{p(\boldsymbol{X}_i,0)}{p(\boldsymbol{X}_i,s)}\frac{\hat{p}(\boldsymbol{X}_i,0)}{p(\boldsymbol{X}_i,0)}\frac{p(\boldsymbol{X}_i,s)}{\hat{p}_s(\boldsymbol{X}_i,s)}\right]\\ &=\frac{e_a(\boldsymbol{X}_i,s)}{\hat{e}_a(\boldsymbol{X}_i,s)}\frac{\hat{p}(\boldsymbol{X}_i,0)}{p(\boldsymbol{X}_i,0)}\frac{p(\boldsymbol{X}_i,s)}{\hat{p}(\boldsymbol{X}_i,s)}\left[\frac{I(A_i=a)}{e_a(\boldsymbol{X}_i,s)}\frac{p(\boldsymbol{X}_i,0)}{p(\boldsymbol{X}_i,s)}\right]\\ &=\hat{\theta}_i(a,s)w_i(a,s)\end{aligned}$$

which proves (i) and (ii).

□

Sub-Lemma 1.2 *For a fixed number of studies m,*

$$\hat{\theta}_i(a,s) \xrightarrow{\mathbb{P}} 1$$

for each $s = 1,2,...,m$ and $a \in \mathcal{A}$ as min$\{\tilde{\boldsymbol{n}}\} \to \infty$.

<u>Proof of Sub-Lemma 1.2</u>: From Slutkzy's Theorem, we have that

$$\begin{aligned}&\frac{e_a(\boldsymbol{X}_i,s)}{\hat{e}_a(\boldsymbol{X}_i,s)} \xrightarrow{\mathbb{P}} 1\\ &\frac{\hat{p}(\boldsymbol{X}_i,0)}{p(\boldsymbol{X}_i,0)}\frac{p(\boldsymbol{X}_i,s)}{\hat{p}(\boldsymbol{X}_i,s)} = \frac{\frac{\hat{p}(\boldsymbol{X}_i,0)}{\hat{p}(\boldsymbol{X}_i,s)}}{\frac{p(\boldsymbol{X}_i,0)}{p(\boldsymbol{X}_i,s)}} \xrightarrow{\mathbb{P}} 1\end{aligned}$$

as $\min\{\tilde{\boldsymbol{n}}\} \to \infty$. We then have

$$\hat{\theta}_i(a,s) \xrightarrow{\mathbb{P}} 1$$

as $\min\{\tilde{\boldsymbol{n}}\} \to \infty$.

□

Sub-Lemma 1.3 *For fixed m,*

(i) $\forall \epsilon > 0,\ P(M_n(a,s) > \epsilon) \to 0$

(ii) $\forall \epsilon > 0,\ P(M_n^*(a,s) > \epsilon) \to 0$

as min$\{\tilde{\boldsymbol{n}}\} \to \infty$ and $a \in \mathcal{A}$, where

$$\begin{aligned}M_n(a,s) &= max\left\{\left|C_{[1]}(a,s)\right|, \left|C_{[n_s]}(a,s)\right|\right\}\\ M_n^*(a,s) &= max\left\{\left|C^*_{[1]}(a,s)\right|, \left|C^*_{[n_s]}(a,s)\right|\right\}\end{aligned}$$

and for all $j = 1,2,...,n_s$

$$\begin{aligned}C_{[j]}(a,s) &= \frac{1}{n_s}\sum_{i:S_i=s}\hat{\theta}_{[j]}(a,s)w_i(a,s) - E\left(w(a,s)|S=s\right)\\ C^*_{[j]}(a,s) &= \frac{1}{n_s}\sum_{i:S_i=s}\hat{\theta}_{[j]}(a,s)w_i(a,s)Y_i - E\left(w(l,s)Y|S=s\right)\end{aligned}$$

and $\hat{\theta}_{[j]}(a,s)$ *is the* j*th largest* $\hat{\theta}_i(a,s)$ *for* $i \in \{1,2,...,n : S_i = s\}$.

Proof of Sub-Lemma 2.3: For each $a \in \mathcal{A}$ and $s = 1,2,...,m$,

$$\frac{1}{n_s}\sum_{i:S_i=s} w_i(a,s) \xrightarrow{\mathbb{P}} E\left(w(a,s)|S=s\right)$$

and

$$\frac{1}{n_s}\sum_{i:S_i=s} w_i(a,s)Y_i \xrightarrow{\mathbb{P}} E\left(w(a,s)Y|S=s\right)$$

as $\min\{\tilde{\boldsymbol{n}}\} \to \infty$ under Condition (i) and the WWLN. By Sub-Lemma 1.2 and Slutzky's Theorem, we have for $j = 1,2,...,n_s$,

$$\begin{aligned}
&\hat{\theta}_{[j]}(a,s)\left[\frac{1}{n_s}\sum_{i:S_i=s} w_i(a,s)\right] \xrightarrow{\mathbb{P}} E\left(w(a,s)|S=s\right)\\
&\Rightarrow C_{[j]}(l,s) \xrightarrow{\mathbb{P}} 0\\
&\Rightarrow P\left(\left|C_{[j]}(l,s)\right| > \epsilon\right) \to 0 \text{ for all } \epsilon > 0
\end{aligned} \tag{13}$$

as $\min\{\tilde{\boldsymbol{n}}\} \to \infty$. Similarly, we have

$$P\left(\left|C_{[j]}(a,s)\right| > \epsilon\right) \to 0 \text{ for all } \epsilon > 0 \tag{14}$$

as $\min\{\tilde{\boldsymbol{n}}\} \to \infty$. Since Equations (13) and (14) apply for all j, we have that

$$\begin{aligned}
&P(M_n(a,s) > \epsilon) \to 0\\
&P(M_n^*(a,s) > \epsilon) \to 0
\end{aligned}$$

as $\min\{\tilde{\boldsymbol{n}}\} \to \infty$ for all $\epsilon > 0$.

□

Lemma 2: *Assume that the models specified in Equation 1 and Equation 2 are correct, Conditions (i)-(iv) and (vii)-(xi) hold, and* $m = o\left(\frac{n}{log(n)}\right)$*, so the number of studies grows with the sample size at a bounded rate. Then,*

(i) $||\hat{\boldsymbol{\theta}}_n - \boldsymbol{\theta}_0|| \xrightarrow{\mathbb{P}} 0$

(ii) $||\hat{\boldsymbol{\beta}}_n - \boldsymbol{\beta}_0|| \xrightarrow{\mathbb{P}} 0$

as $n, m \to \infty$ *where* $\hat{\boldsymbol{\theta}}_n$ *is the MLE of* $\boldsymbol{\theta}_0$ *and* $\hat{\boldsymbol{\beta}}_n$ *is the MLE of* $\boldsymbol{\beta}_0$.

Proof of Lemma 2: From Sub-Lemmas 2.1 and 2.2, we have that $||\hat{\boldsymbol{\theta}}_n - \boldsymbol{\theta}_0||^2 = O_P\left(\frac{m}{n}\right)$ and $||\hat{\boldsymbol{\beta}}_n - \boldsymbol{\beta}_0||^2 = O_P\left(\frac{m}{n}\right)$ respectively. Because $m = o\left(\frac{n}{\log(n)}\right)$, we know that for all $C > 0$, there exists an integer $n_0 \geq 1$ such that

$$m < c\frac{n}{\log(n)}$$

for all $n \geq n_0$. Notice that for $n \geq 3$, $\log(n) > 1$ and $\frac{n}{\log(n)} < n$. So, for all $n \geq n_1 = \max\{3, n_0\}$,

$$m < C\frac{n}{\log(n)} < Cn$$

for all $n > n_1$. Therefore,

$$\begin{aligned} & m = o(n) \\ \Rightarrow & \frac{m}{n} = o(1) \\ \Rightarrow & \frac{m}{n} = o_P(1). \end{aligned} \tag{15}$$

Then, we have that as $n, m \to \infty$ with $m = o\left(\frac{n}{\log(n)}\right)$

$$\begin{aligned} & ||\hat{\boldsymbol{\theta}}_n - \boldsymbol{\theta}_0||^2 = O_P\left(\frac{m}{n}\right);\ \textit{by Sub-Lemma 2.1} \\ \Rightarrow & \frac{n}{m}||\hat{\boldsymbol{\theta}}_n - \boldsymbol{\theta}||^2 = O_P(1) \\ \Rightarrow & ||\hat{\boldsymbol{\theta}}_n - \boldsymbol{\theta}_0||^2 = \frac{m}{n}\frac{n}{m}||\hat{\boldsymbol{\theta}}_n - \boldsymbol{\theta}_0||^2 = o_P(1)O_P(1);\ \textit{by Equation 15} \\ \Rightarrow & ||\hat{\boldsymbol{\theta}}_n - \boldsymbol{\theta}_0||^2 = o_P(1) \\ \Rightarrow & ||\hat{\boldsymbol{\theta}}_n - \boldsymbol{\theta}_0||^2 \xrightarrow{\mathbb{P}} 0 \end{aligned} \tag{16}$$

and we can similarly show that as $n, m \to \infty$ with $m = o\left(\frac{n}{\log(n)}\right)$

$$||\hat{\boldsymbol{\beta}}_n - \boldsymbol{\beta}_0||^2 \xrightarrow{\mathbb{P}} 0$$

because we have that $||\hat{\boldsymbol{\beta}}_n - \boldsymbol{\beta}_0||^2 = O_P\left(\frac{m}{n}\right)$ from Sub-Lemma 2.2.

□

Sub-Lemma 2.1: *If the models in Equation 1 are correctly specified, Conditions (i), (iii), (vii), (viii), and (x) hold, then*

$$||\hat{\boldsymbol{\theta}}_n - \boldsymbol{\theta}_0||^2 = O_P\left(\frac{m}{n}\right)$$

if the number of studies, m, grows with the sample size, n, with $m = o\left(\frac{n}{log(n)}\right)$ *by Theorem 2.1 of He and Shao [1], where* $\hat{\boldsymbol{\theta}}_n$ *is the MLE of* $\boldsymbol{\theta}_0$.

Proof of Sub-Lemma 2.1: Convergence results for M-estimators, such as MLEs for parameters of GLMs, when the number of parameters grows with the sample size are provided by He and Shao [1]. Because we were able to write our m study-specific treatment models in Equation 1 as a single model with $m \times q$ parameters contained in $\boldsymbol{\theta}_0$ where m grows with n, we only to confirm the conditions of He and Shao [1] Theorem 2.1 hold to prove Sub-Lemma 2.1. We do this by following Example 3 in He and Shao [1].

First, we confirm that $\hat{\theta}_n$ is a minimizer of $\sum_{i=1}^n \rho((A_i, \boldsymbol{V}_i), \boldsymbol{\theta})$ over $\boldsymbol{\theta} \in \mathbb{R}^{qm}$ for some function $\rho((A_i, \boldsymbol{V}_i), \boldsymbol{\theta})$ that is convex in θ. For the model shown in Equation 1, the log-likelihood is

$$\begin{aligned}
\log(L(\boldsymbol{\theta})) =& \log\left[\prod_{i=1}^n \left(\frac{\exp\{\boldsymbol{Z}_i^t\boldsymbol{\theta}\}}{1+\exp\{\boldsymbol{Z}_i^t\boldsymbol{\theta}\}}\right)^{I(A_i=a)} \left(\frac{1}{1+\exp\{\boldsymbol{Z}_i^t\boldsymbol{\theta}\}}\right)^{1-I(A_i=a)}\right] \\
=& \log\left[\prod_{i=1}^n \frac{(\exp\{\boldsymbol{Z}_i^t\boldsymbol{\theta}\})^{I(A_i=a)}}{1+\exp\{\boldsymbol{Z}_i^t\boldsymbol{\theta}\}}\right] \\
=& \sum_{i=1}^n \left[I(A_i=a)\log\left(\exp\{\boldsymbol{Z}_i^t\boldsymbol{\theta}_0\}\right) - \log\left(1+\exp\{\boldsymbol{Z}_i^t\boldsymbol{\theta}\}\right)\right] \\
=& \sum_{i=1}^n \left[I(A_i=a)\left(\boldsymbol{Z}_i^t\boldsymbol{\theta}\right) - \log\left(1+\exp\{\boldsymbol{Z}_i^t\boldsymbol{\theta}\}\right)\right].
\end{aligned}$$

Let $\rho((A_i, \boldsymbol{V}_i), \boldsymbol{\theta}) = I(A_i = a)\left(\boldsymbol{Z}_i^t\boldsymbol{\theta}\right) - \log\left(1+\exp\{\boldsymbol{Z}_i^t\boldsymbol{\theta}\}\right)$ which is convex in $\boldsymbol{\theta}$ [1]. By the definition of MLEs, $\hat{\boldsymbol{\theta}}_n$ minimizes $\sum_{i=1}^n \rho((A_i, \boldsymbol{V}_i), \boldsymbol{\theta})$ over $\boldsymbol{\theta} \in \mathbb{R}^{qm}$. Letting $\psi((A_i, \boldsymbol{V}_i), \boldsymbol{\theta}) \equiv \frac{\partial}{\partial \boldsymbol{\theta}}\rho((A_i, \boldsymbol{V}_i), \boldsymbol{\theta})$, we have

$$\begin{aligned}
\psi((A_i, \boldsymbol{V}_i), \boldsymbol{\theta}) =& \frac{\partial}{\partial \boldsymbol{\theta}}\left[I(A_i=a)\left(\boldsymbol{Z}_i^t\boldsymbol{\theta}\right) - \log\left(1+\exp\{\boldsymbol{Z}_i^t\boldsymbol{\theta}\}\right)\right] \\
=& \left(I(A_i=a) - \frac{\exp\{\boldsymbol{Z}_i^t\boldsymbol{\theta}\}}{1+\exp\{\boldsymbol{Z}_i^t\boldsymbol{\theta}\}}\right)\boldsymbol{Z}_i
\end{aligned} \tag{17}$$

We now verify the six conditions He and Shao [1] assumed held when proving Theorem 2.1. The first condition is:

R0. $||\sum_{i=1}^n \psi((A_i, \boldsymbol{V}_i), \hat{\boldsymbol{\theta}}_n)|| = o_P(n^{\frac{1}{2}})$

Because ρ is differentiable with respect to $\boldsymbol{\theta}$, Condition R0 is met [1].

The second condition is:

R1. $\exists C$ and $r \in (0, 2]$ such that

$$\max_{i \leq n}\left\{E_\theta\left[\sup_{\boldsymbol{\tau}:||\boldsymbol{\tau}-\boldsymbol{\theta}||\leq d}\left(||\eta_i(\boldsymbol{\tau}, \boldsymbol{\theta})||^2\right)\right]\right\} \leq n^C d^r$$

for $0 < d \leq 1$ where

$$\begin{aligned}
\eta_i(\boldsymbol{\tau}, \boldsymbol{\theta}) =& \psi((A_i, \boldsymbol{V}_i), \boldsymbol{\tau}) - \psi((A_i, \boldsymbol{V}_i), \boldsymbol{\theta}) \\
& - E\left[\psi((A_i, \boldsymbol{V}_i), \boldsymbol{\tau})\right] + E\left[\psi((A_i, \boldsymbol{V}_i), \boldsymbol{\theta})\right]
\end{aligned}$$

To show that Condition R1 holds, notice that

$$||\boldsymbol{Z}_i||^2 = \boldsymbol{Z}_i^t\boldsymbol{Z}_i = \sum_{s\in\mathcal{S}} \boldsymbol{Z}_{is}^t\boldsymbol{Z}_{is} = \sum_{s\in\mathcal{S}} I(S_i = s)\boldsymbol{V}_i^t\boldsymbol{V}_i = \boldsymbol{V}_i^t\boldsymbol{V}_i = ||\boldsymbol{V}_i||^2 \tag{18}$$

and, following from Equation 17,

$$\eta_i(\boldsymbol{\tau},\boldsymbol{\theta}) = \left(\frac{\exp\{\boldsymbol{Z}_i^t\boldsymbol{\theta}\}}{1+\exp\{\boldsymbol{Z}_i^t\boldsymbol{\theta}\}} - \frac{\exp\{\boldsymbol{Z}_i^t\boldsymbol{\tau}\}}{1+\exp\{\boldsymbol{Z}_i^t\boldsymbol{\tau}\}}\right)\boldsymbol{Z}_i + E\left[\left(\frac{\exp\{\boldsymbol{Z}_i^t\boldsymbol{\tau}\}}{1+\exp\{\boldsymbol{Z}_i^t\boldsymbol{\tau}\}} - \frac{\exp\{\boldsymbol{Z}_i^t\boldsymbol{\theta}\}}{1+\exp\{\boldsymbol{Z}_i^t\boldsymbol{\theta}\}}\right)\boldsymbol{Z}_i\right].$$

The first derivative of $\text{expit}(x) = \frac{\exp\{x\}}{1+\exp\{x\}}$ is $\frac{\exp\{x\}}{(1+\exp\{x\})^2}$. For all x, $\exp\{x\} > 0$ and the function $f(y) = \frac{y}{(1+y)^2}$ is bounded below by 0 and above by 0.25 (its global maximum at $y = 1$) for $y > 0$. Therefore, the first derivative of the expit function exists and is bounded, making the expit function a Lipschitz function. Thus, there exists a constant $C_1 > 0$ such that, for all i,

$$\begin{aligned}\left|\frac{\exp\{\boldsymbol{Z}_i^t\boldsymbol{\theta}\}}{1+\exp\{\boldsymbol{Z}_i^t\boldsymbol{\theta}\}} - \frac{\exp\{\boldsymbol{Z}_i^t\boldsymbol{\tau}\}}{1+\exp\{\boldsymbol{Z}_i^t\boldsymbol{\tau}\}}\right| &\le C_1\left|\boldsymbol{Z}_i^t\boldsymbol{\theta} - \boldsymbol{Z}_i^t\boldsymbol{\tau}\right| \\ &\le C_1||\boldsymbol{Z}_i||||\boldsymbol{\theta}-\boldsymbol{\tau}||;\ \textit{Cauchy-Schwarz} \\ &= C_1||\boldsymbol{V}_i||||\boldsymbol{\theta}-\boldsymbol{\tau}||;\ \textit{Equation 18.}\end{aligned}$$

So, for all i,

$$\begin{aligned}\left|\left|\left(\frac{\exp\{\boldsymbol{Z}_i^t\boldsymbol{\theta}\}}{1+\exp\{\boldsymbol{Z}_i^t\boldsymbol{\theta}\}} - \frac{\exp\{\boldsymbol{Z}_i^t\boldsymbol{\tau}\}}{1+\exp\{\boldsymbol{Z}_i^t\boldsymbol{\tau}\}}\right)\boldsymbol{Z}_i\right|\right| &= \left|\frac{\exp\{\boldsymbol{Z}_i^t\boldsymbol{\theta}\}}{1+\exp\{\boldsymbol{Z}_i^t\boldsymbol{\theta}\}} - \frac{\exp\{\boldsymbol{Z}_i^t\boldsymbol{\tau}\}}{1+\exp\{\boldsymbol{Z}_i^t\boldsymbol{\tau}\}}\right|||\boldsymbol{Z}_i|| \\ &\le C_1||\boldsymbol{V}_i||^2||\boldsymbol{\theta}-\boldsymbol{\tau}||\end{aligned} \tag{19}$$

Let $M_i = \max\{||\boldsymbol{V}_i||^2, E(||\boldsymbol{V}_i||^2)\} = \max\{||\boldsymbol{Z}_i||^2, E(||\boldsymbol{Z}_i||^2)\}$ and notice that $M_i \ge 0$. Then, from Equation 19 and Jensen's inequality we have

$$\begin{aligned}||\eta_i(\boldsymbol{\tau},\boldsymbol{\theta})|| &\le C_1||\boldsymbol{V}_i||^2||\boldsymbol{\tau}-\boldsymbol{\theta}|| + E\left(C_1||\boldsymbol{V}_i||^2||\boldsymbol{\tau}-\boldsymbol{\theta}||\right) \\ &= C_1\left[||\boldsymbol{V}_i||^2 + E\left(||\boldsymbol{V}_i||^2\right)\right]||\boldsymbol{\tau}-\boldsymbol{\theta}||\end{aligned}$$

and then that

$$||\eta_i(\boldsymbol{\tau},\boldsymbol{\theta})|| \le 2C_1M_i||\boldsymbol{\tau}-\boldsymbol{\theta}|| \tag{20}$$

Notice that $\left|\left|\frac{\boldsymbol{Z}_i^t}{||\boldsymbol{Z}_i||}\right|\right| = 1$ so by Assumption (viii), letting $\boldsymbol{\alpha} = \boldsymbol{\gamma} = \frac{\boldsymbol{Z}_i^t}{||\boldsymbol{Z}_i||}$,

$$\sum_{i=1}^n \left|\frac{\boldsymbol{Z}_i^t}{||\boldsymbol{Z}_i||}\boldsymbol{Z}_i\right|^2\left|\frac{\boldsymbol{Z}_i^t}{||\boldsymbol{Z}_i||}\boldsymbol{Z}_i\right|^2 = \sum_{i=1}^n ||\boldsymbol{Z}_i||^4 = O(n) \tag{21}$$

By Jensen' inequality and the linearity of expectations, we then have that

$$\sum_{i=1}^n E\left(||\boldsymbol{Z}_i||^2\right)^2 \le \sum_{i=1}^n E\left(||\boldsymbol{Z}_i||^4\right) = E\left(\sum_{i=1}^n ||\boldsymbol{Z}_i||^4\right) = O(n). \tag{22}$$

Because $M_i \ge 0$, $M_i^2 = \max\left\{||\boldsymbol{Z}_i||^4, E\left(||\boldsymbol{Z}_i||^2\right)^2\right\}$, so by Equations 21 and 22 we have that

$$\sum_{i=1}^n M_i^2 = O(n) \tag{23}$$

and then we have that

$$\begin{aligned} M_i^2 &\leq \sum_{i=1}^{n} M_i^2 = O(n) \\ \Rightarrow M_i &= O\left(n^{\frac{1}{2}}\right) \end{aligned} \tag{24}$$

From Example 1 in [1], we then have that Condition R1 holds because

$$||\eta_i(\boldsymbol{\tau}, \boldsymbol{\theta})|| \leq 2C_1 M_i ||\boldsymbol{\tau} - \boldsymbol{\theta}|| \tag{25}$$

where $M_i = O\left(n^{r_1}\right)$ for some $r_1 > 0$.

The third condition is:

R2. $\sum_{i=1}^{n} E\left(||\psi((A_i, \boldsymbol{V}_i), \boldsymbol{\theta}_0)||^2\right) = O(nmq)$

Condition R2 holds because

$$\begin{aligned} \sum_{i=1}^{n} ||\psi((A_i, \boldsymbol{V}_i).\boldsymbol{\theta}_0)||^2 &= \sum_{i=1}^{n} \left(I(A_i = a) - \frac{\exp\{\boldsymbol{Z}_i^t \boldsymbol{\theta}_0\}}{1 + \exp\{\boldsymbol{Z}_i^t \boldsymbol{\theta}_0\}} \right)^2 ||\boldsymbol{Z}_i||^2; \ \textit{Equation 17} \\ &\leq \sum_{i=1}^{n} ||\boldsymbol{Z}_i||^2 \\ &= \sum_{i=1}^{n} ||\boldsymbol{V}_i||^2; \ \textit{Equation 18} \end{aligned}$$

because $-1 < I(A_i = a) - \frac{\exp\{\boldsymbol{Z}_i^t \boldsymbol{\theta}_0\}}{1+\exp\{\boldsymbol{Z}_i^t \boldsymbol{\theta}_0\}} < 1$. So, under Assumption (vii) we have

$$\sum_{i=1}^{n} E\left(||\psi((A_i, \boldsymbol{X}_i), \boldsymbol{\theta}_0)||^2\right) \leq E\left(\sum_{i=1}^{n} ||\boldsymbol{V}_i||^2\right) = O(nm)$$

and since q is constant, $O(nm) = O(nmq)$.

The fourth condition is:

R3. There exists a sequence of $(mq) \times (mq)$ matrices, $\boldsymbol{D}_n$ with

$$\lim\ \inf_{n \to \infty} \lambda_{min}(\boldsymbol{D}_n) > 0$$

such that for any $B > 0$ and uniformly in $\boldsymbol{\alpha} \in H_m = \{\boldsymbol{\alpha} \in \mathbb{R}^m : ||\boldsymbol{\alpha}|| = 1\}$

$$\sup_{||\boldsymbol{\theta} - \boldsymbol{\theta}_0|| \leq B\left(\frac{qm}{n}\right)^{\frac{1}{2}}} \left| \boldsymbol{\alpha}^t \sum_{i=1}^{n} E_{\boldsymbol{\theta}_0}\left[\psi((A_i, \boldsymbol{V}_i), \boldsymbol{\theta}) - \psi((A_i, \boldsymbol{V}_i), \boldsymbol{\theta}_0)\right] - n\boldsymbol{\alpha}^t \boldsymbol{D}_n(\boldsymbol{\theta} - \boldsymbol{\theta}_0) \right|$$

is $o(n^{\frac{1}{2}})$.

Let $\boldsymbol{D}_n = \frac{1}{n}\sum_{i=1}^{n} \frac{\exp\{\boldsymbol{Z}_i^t\boldsymbol{\theta}_0\}}{1+\exp\{\boldsymbol{Z}_i^t\boldsymbol{\theta}_0\}}\boldsymbol{Z}_i\boldsymbol{Z}_i^t$. Because

$$\boldsymbol{Z}_i\boldsymbol{Z}_i^t = \text{blockdiag}\left(I(S_i=1)\boldsymbol{V}_i\boldsymbol{V}_i^t, ..., I(S_i=m)\boldsymbol{V}_i\boldsymbol{V}_i^t\right)$$

and $\boldsymbol{Z}_i^t\boldsymbol{\theta}_0 = \boldsymbol{V}_i^t\boldsymbol{\theta}_{0,S_i}$, we can write

$$\boldsymbol{D}_n = \text{block}\left(\boldsymbol{D}_{n,1}, ..., \boldsymbol{D}_{n,m}\right)$$

where

$$\boldsymbol{D}_{n,s} = \frac{1}{n}\sum_{i=1}^{n} I(S_i=s)\frac{\exp\{\boldsymbol{V}_i^t\boldsymbol{\theta}_{0,s}\}}{1+\exp\{\boldsymbol{V}_i^t\boldsymbol{\theta}_{0,s}\}}\boldsymbol{V}_i^t\boldsymbol{V}_i.$$

The eigenvalues of a block diagonal matrix are the eigenvalues of each block, so by Assumption (x), we have that $\lim\inf_{n\to\infty}\lambda_{min}(\boldsymbol{D}_n) > 0$. Then, from Example 3 in [1] we have that Condition R3 holds under Assumptions (vii) and (viii).

The fifth condition is:

R4. For any $\boldsymbol{\theta} \in \mathbb{R}^{qm}$, $\boldsymbol{\alpha} \in H_{qm}$, and $B > 0$,

$$\sup_{\boldsymbol{\tau}:||\boldsymbol{\tau}-\boldsymbol{\theta}||\leq B\left(\frac{mq}{n}\right)^{\frac{1}{2}}} \left\{\sum_{i=1}^{n} E_{\boldsymbol{\theta}}\left[|\boldsymbol{\alpha}^t\eta_i(\boldsymbol{\tau},\boldsymbol{\theta})|^2\right]\right\} = O(m)$$

To see that Condition R4 holds, first notice, by Cauchy–Schwarz and Equation 20 we have that there exists some constant $C > 0$ such that

$$\begin{aligned}\sum_{i=1}^{n}(\boldsymbol{\alpha}^t\eta_i(\boldsymbol{\tau},\boldsymbol{\theta}))^2 \leq& \sum_{i=1}^{n}||\boldsymbol{\alpha}||^2||\eta_i(\boldsymbol{\tau},\boldsymbol{\theta})||^2 = \sum_{i=1}^{n}||\eta_i(\boldsymbol{\tau},\boldsymbol{\theta})||^2\\ \leq& C||\boldsymbol{\tau}-\boldsymbol{\theta}||^2\sum_{i=1}^{n}M_i^2\end{aligned}$$

because $\boldsymbol{\alpha} \in H_{qm}$. So, there exists constants $C, C^* > 0$ and n_0 such that for all $n \geq n_0$

$$\begin{aligned}&\sup_{\boldsymbol{\tau}:||\boldsymbol{\tau}-\boldsymbol{\theta}||\leq B\left(\frac{mq}{n}\right)^{\frac{1}{2}}} \Big\{\sum_{i=1}^{n} E_{\boldsymbol{\theta}}\left[|\boldsymbol{\alpha}^t\eta_i(\boldsymbol{\tau},\boldsymbol{\theta})|^2\right]\Big\}\\ &\qquad\leq \sup_{\boldsymbol{\tau}:||\boldsymbol{\tau}-\boldsymbol{\theta}||\leq B\left(\frac{mq}{n}\right)^{\frac{1}{2}}} \left\{C||\boldsymbol{\tau}-\boldsymbol{\theta}||^2 E_{\boldsymbol{\theta}}\left[\sum_{i=1}^{n}M_i^2\right]\right\};\ \textit{by Equation 20}\\ &\qquad\leq CB^2q\left(\frac{m}{n}\right)E_{\boldsymbol{\theta}}\left[\sum_{i=1}^{n}M_i^2\right]\\ &\qquad\leq C^*\left(\frac{m}{n}\right)n;\ \textit{by Equation 23 and because } q \textit{ is a constant}\\ &\qquad= C^*m\end{aligned}$$

which proves Condition R4.

The sixth, and final, condition is:

R5. For any $\boldsymbol{\theta} \in \mathbb{R}^{mq}$ and $B > 0$,

$$\sup_{\boldsymbol{\alpha} \in H_{qm}} \left\{ \sup_{\boldsymbol{\tau}:||\boldsymbol{\tau}-\boldsymbol{\theta}|| \leq B\left(\frac{qm}{n}\right)^{\frac{1}{2}}} \sum_{i=1}^{n} \left[\boldsymbol{\alpha}^t \eta_i(\boldsymbol{\tau}, \boldsymbol{\theta})\right]^2 \right\} = O_P(m)$$

Using the same logic as in the proof that Condition R4 holds, we have that there exists a constant $C^* > 0$ and an n_0 such that for all $n > n_0$

$$\begin{aligned}\sup_{\boldsymbol{\alpha} \in H_{qm}} \left\{ \sup_{\boldsymbol{\tau}:||\boldsymbol{\tau}-\boldsymbol{\theta}|| \leq B\left(\frac{mq}{n}\right)^{\frac{1}{2}}} \sum_{i=1}^{n} \left[\boldsymbol{\alpha}^t \eta_i(\boldsymbol{\tau}, \boldsymbol{\theta})\right]^2 \right\} \leq & CB^2 \left(\frac{mq}{n}\right) \sum_{i=1}^{n} M_i^2 \\ \leq & C^* \left(\frac{m}{n}\right) n = C^* m\end{aligned}$$

which proves Condition R5 holds because, for all $n > n_0$,

$$P\left(\frac{1}{m} \sup_{\boldsymbol{\alpha} \in H_{qm}} \left\{ \sup_{\boldsymbol{\tau}:||\boldsymbol{\tau}-\boldsymbol{\theta}|| \leq B\left(\frac{mq}{n}\right)^{\frac{1}{2}}} \sum_{i=1}^{n} \left[\boldsymbol{\alpha}^t \eta_i(\boldsymbol{\tau}, \boldsymbol{\theta})\right]^2 \right\} > C^* \right) = 0.$$

□

Sub-Lemma 2.2: *If the model in Equation 2 is correctly specified, Conditions (i)-(iv), (vii), (ix), and (xi) hold,*

$$||\hat{\boldsymbol{\beta}}_n - \boldsymbol{\beta}_0||^2 = O_P\left(\frac{m}{n}\right)$$

if the number of studies, m, grows with the sample size, n, with $m = o\left(\frac{n}{log(n)}\right)$ *by Theorem 2.1 of [1], where* $\hat{\beta}_n$ *is the MLE of* $\boldsymbol{\beta}_0$.

<u>Proof of Sub-Lemma 2.2</u>: Our study membership model specified in Equation 2 has $m \times q$ parameters parameters contained in $\boldsymbol{\beta}_0$ where m grows with n. We only to confirm the conditions of He and Shao [1] Theorem 2.1 hold to prove Sub-Lemma 2.2 and therefore this proof follows the proof of Sub-Lemma 2.1.

First, we confirm that $\hat{\beta}_n$ is a minimizer of $\sum_{i=1}^{n} \rho((S_i, \boldsymbol{V}_i), \boldsymbol{\beta})$ over $\boldsymbol{\beta} \in \mathbb{R}^{qm}$ for some function $\rho((S_i, \boldsymbol{V}_i), \boldsymbol{\beta})$ that is convex in β. For the model shown in Equation 2, the log-likelihood is

$$\begin{aligned}\log(L(\boldsymbol{\beta})) =& \log\left[\prod_{i=1}^{n} \prod_{s \in \{0, \mathcal{S}\}} P(S_i = s|\boldsymbol{X}_i)^{I(S_i=s)}\right] \\ =& \log\left[\prod_{i=1}^{n} \prod_{s \in \mathcal{S}} \left(\frac{1}{1 + \sum_{k=1}^{m} \exp\{\boldsymbol{U}_{ik}^t \boldsymbol{\beta}\}}\right)^{\sum_{s \in \{0, \mathcal{S}\}} I(S_i=s)} \exp\{\boldsymbol{U}_{is}^t \boldsymbol{\beta}\}^{I(S_i=s)}\right] \\ =& \sum_{i=1}^{n} \sum_{s \in \mathcal{S}} \left[I(S_i = s)\boldsymbol{U}_{is}^t \boldsymbol{\beta} - \log\left(1 + \sum_{k \in \mathcal{S}} \exp\{\boldsymbol{U}_{ik}^t \boldsymbol{\beta}\}\right)\right] \\ =& \sum_{i=1}^{n} \left\{ \left[\sum_{s \in \mathcal{S}} I(S_i = s)\boldsymbol{U}_{is}^t\right] \boldsymbol{\beta} - \log\left(1 + \sum_{k \in \mathcal{S}} \exp\{\boldsymbol{U}_{ik}^t \boldsymbol{\beta}\}\right) \right\}\end{aligned}$$

because $\sum_{s\in\{0,\mathcal{S}\}} I(S_i = s) = 1$. Let

$$\rho((S_i, \boldsymbol{V}_i), \boldsymbol{\theta}) = \left[\sum_{s\in\mathcal{S}} I(S_i = s)\boldsymbol{U}_{is}^t\right]\boldsymbol{\beta} - \log\left(1 + \sum_{s\in\mathcal{S}} \exp\{\boldsymbol{U}_{is}^t\boldsymbol{\beta}\}\right).$$

which is convex in $\boldsymbol{\beta}$ [1]. By the definition of MLEs, $\hat{\boldsymbol{\beta}}_n$ minimizes $\sum_{i=1}^n \rho((S_i, \boldsymbol{V}_i), \boldsymbol{\beta})$ over $\boldsymbol{\beta} \in \mathbb{R}^{qm}$. Letting $\psi((S_i, \boldsymbol{V}_i), \boldsymbol{\beta}) = \frac{\partial}{\partial\boldsymbol{\beta}}\rho((S_i, \boldsymbol{V}_i), \boldsymbol{\beta})$, we have

$$\begin{aligned}
\psi((S_i, \boldsymbol{V}_i), \boldsymbol{\beta}) =& \sum_{s\in\mathcal{S}} I(S_i = s)\boldsymbol{U}_{is} - \sum_{s\in\mathcal{S}} \frac{\exp\{\boldsymbol{U}_{is}^t\boldsymbol{\beta}\}}{1 + \sum_{k\in\mathcal{S}} \exp\{\boldsymbol{U}_{ik}^t\boldsymbol{\beta}\}}\boldsymbol{U}_{is} \\
=& \sum_{s\in\mathcal{S}} \left[I(S_i = s) - \frac{\exp\{\boldsymbol{U}_{is}^t\boldsymbol{\beta}\}}{1 + \sum_{k\in\mathcal{S}} \exp\{\boldsymbol{U}_{ik}^t\boldsymbol{\beta}\}}\right]\boldsymbol{U}_{is}.
\end{aligned} \tag{26}$$

We now verify the six conditions He and Shao [1] assumed held when proving Theorem 2.1. The first condition is:

R0. $||\sum_{i=1}^n \psi((S_i, \boldsymbol{V}_i), \hat{\boldsymbol{\beta}}_n)|| = o_P(n^{\frac{1}{2}})$

Because ρ is differentiable with respect to $\boldsymbol{\beta}$, Condition R0 is met [1].

The second condition is

R1. $\exists C$ and $r \in (0, 2]$ such that

$$\max_{i\le n}\left\{E_\theta\left[\sup_{\boldsymbol{\tau}:||\boldsymbol{\tau}-\boldsymbol{\theta}||\le d}\left(||\eta_i(\boldsymbol{\tau}, \boldsymbol{\beta})||^2\right)\right]\right\} \le n^C d^r$$

for $0 < d \le 1$ where

$$\begin{aligned}
\eta_i(\boldsymbol{\tau}, \boldsymbol{\theta}) =& \psi((S_i, \boldsymbol{V}_i), \boldsymbol{\tau}) - \psi((S_i, \boldsymbol{V}_i), \boldsymbol{\theta}) \\
&- E\left[\psi((S_i, \boldsymbol{V}_i), \boldsymbol{\tau})\right] + E\left[\psi((S_i, \boldsymbol{V}_i), \boldsymbol{\theta})\right]
\end{aligned}$$

To show that Condition R1 holds, recall that for each $i = 1, 2, ..., n$ and $s \in \mathcal{S}$, $\boldsymbol{U}_{is} = e_s \otimes \boldsymbol{V}_i \in \mathbb{R}^{mq}$ where e_s is the sth column of the $m \times m$ identity matrix, so $||\boldsymbol{U}_{i,s}|| = ||\boldsymbol{V}_i||$ and $\boldsymbol{U}_{is}^t\boldsymbol{\beta} = \boldsymbol{V}_i^t\boldsymbol{\beta}_s$ for each $i = 1, 2, ..., n$ and $s \in \mathcal{S}$. Therefore,

$$\begin{aligned}
&\Big|\Big|\sum_{s\in\mathcal{S}}\left(\frac{\exp\{\boldsymbol{U}_{is}^t\boldsymbol{\beta}\}}{1 + \sum_{k=1}^m \exp\{\boldsymbol{U}_{ik}^t\boldsymbol{\beta}\}} - \frac{\exp\{\boldsymbol{U}_{is}^t\boldsymbol{\tau}\}}{1 + \sum_{k=1}^m \exp\{\boldsymbol{U}_{ik}^t\boldsymbol{\tau}\}}\right)\boldsymbol{U}_{is}\Big|\Big| \\
&\le \sum_{s\in\mathcal{S}}\left|\left|\left(\frac{\exp\{\boldsymbol{U}_{is}^t\boldsymbol{\beta}\}}{1 + \sum_{k=1}^m \exp\{\boldsymbol{U}_{ik}^t\boldsymbol{\beta}\}} - \frac{\exp\{\boldsymbol{U}_{is}^t\boldsymbol{\tau}\}}{1 + \sum_{k=1}^m \exp\{\boldsymbol{U}_{ik}^t\boldsymbol{\tau}\}}\right)\boldsymbol{U}_{is}\right|\right| \\
&= \sum_{s\in\mathcal{S}}\left|\left(\frac{\exp\{\boldsymbol{U}_{is}^t\boldsymbol{\beta}\}}{1 + \sum_{k=1}^m \exp\{\boldsymbol{U}_{ik}^t\boldsymbol{\beta}\}} - \frac{\exp\{\boldsymbol{U}_{is}^t\boldsymbol{\tau}\}}{1 + \sum_{k=1}^m \exp\{\boldsymbol{U}_{ik}^t\boldsymbol{\tau}\}}\right)\right| ||\boldsymbol{U}_{is}|| \\
&= ||\boldsymbol{V}_i|| \sum_{s\in\mathcal{S}}\left|\left(\frac{\exp\{\boldsymbol{V}_i^t\boldsymbol{\beta}_s\}}{1 + \sum_{k=1}^m \exp\{\boldsymbol{V}_i^t\boldsymbol{\beta}_k\}} - \frac{\exp\{\boldsymbol{V}_i^t\boldsymbol{\tau}_s\}}{1 + \sum_{k=1}^m \exp\{\boldsymbol{V}_i^t\boldsymbol{\tau}_k\}}\right)\right|
\end{aligned} \tag{27}$$

Consider the following function of $\boldsymbol{x} = (x_1, ..., x_m)^t$

$$\phi_s(\boldsymbol{x}) = \frac{\exp\{x_s\}}{1 + \sum_{k \in S} \exp\{x_k\}}$$

The derivative of $\phi_s(\boldsymbol{x})$ with respect to x_s is

$$\frac{\partial}{\partial x_s}\phi_s(\boldsymbol{x}) = \frac{(1 + \sum_{k \in \mathcal{S}, k \neq s} \exp\{x_k\})\exp\{x_s\}}{\left(1 + \sum_{k \in S} \exp\{x_k\}\right)^2}$$

and the derivative with respect to x_t $t \neq s$ is

$$\begin{aligned}\frac{\partial}{\partial x_t}\phi_s(\boldsymbol{x}) =& \exp\{x_s\}\frac{\partial}{\partial x_t}\frac{1}{1 + \sum_{k \in S, k \neq t} \exp\{x_k\} + \exp\{x_t\}} \\ =& \frac{\exp\{x_s\}\exp\{x_t\}}{\left(1 + \sum_{k \in S} \exp\{x_k\}\right)^2}\end{aligned}$$

All of these partial derivatives exists everywhere and are bounded below and above because $\exp(x) > 0$ for all x. So $\phi_s(\boldsymbol{x})$ is a Lipschitz function and there exists a constant $C > 0$ such that

$$|\phi(\boldsymbol{x}) - \phi(\boldsymbol{y})| \leq C||\boldsymbol{x} - \boldsymbol{y}||$$

This shows that for all $s \in \mathcal{S}$, there exists a constant $C_s > 0$ such that

$$\begin{aligned}&\left|\left(\frac{\exp\{\boldsymbol{V}_i^t\boldsymbol{\beta}_s\}}{1 + \sum_{k \in \mathcal{S}} \exp\{\boldsymbol{V}_i^t\boldsymbol{\beta}_s\}} - \frac{\exp\{\boldsymbol{V}_i^t\boldsymbol{\tau}_s\}}{1 + \sum_{k \in \mathcal{S}} \exp\{\boldsymbol{V}_i^t\boldsymbol{\tau}_s\}}\right)\right| \\ &\qquad \leq C_s \left|\left|\begin{pmatrix}\boldsymbol{V}_i^t(\boldsymbol{\beta}_1 - \boldsymbol{\tau}_1) \\ \vdots \\ \boldsymbol{V}_i^t(\boldsymbol{\beta}_m - \boldsymbol{\tau}_m)\end{pmatrix}\right|\right| \\ &\qquad = C_s \left(\sum_{s \in \mathcal{S}} (\boldsymbol{V}_i^t(\boldsymbol{\beta}_s - \boldsymbol{\tau}_s))^2\right)^{\frac{1}{2}} \\ &\qquad \leq C_s \left(||\boldsymbol{V}_i||^2 \sum_{s \in \mathcal{S}} ||\boldsymbol{\beta}_s - \boldsymbol{\tau}_s||^2\right)^{\frac{1}{2}} \\ &\qquad = C_s \left(||\boldsymbol{V}_i||^2 ||\boldsymbol{\beta} - \boldsymbol{\tau}||^2\right)^{\frac{1}{2}} \\ &\qquad = C_s ||\boldsymbol{V}_i|| ||\boldsymbol{\beta} - \boldsymbol{\tau}||\end{aligned}$$

and that

$$\begin{aligned}&\sum_{s \in \mathcal{S}} \left|\left(\frac{\exp\{\boldsymbol{V}_i^t\boldsymbol{\beta}_s\}}{1 + \sum_{k \in \mathcal{S}} \exp\{\boldsymbol{V}_i^t\boldsymbol{\beta}_s\}} - \frac{\exp\{\boldsymbol{V}_i^t\boldsymbol{\tau}_s\}}{1 + \sum_{k \in \mathcal{S}} \exp\{\boldsymbol{V}_i^t\boldsymbol{\tau}_s\}}\right)\right| \\ &\qquad \leq \left(\sum_{s \in \mathcal{S}} C_s\right) ||\boldsymbol{V}_i|| ||\boldsymbol{\beta} - \boldsymbol{\tau}||\end{aligned} \tag{28}$$

Let $M_i = \max\{||\boldsymbol{V}_i||^2, E(||\boldsymbol{V}_i||^2)\}$ and notice that $M_i \geq 0$. By Equations 27 and 28, we have that there exists a constant $C_1 > 0$

$$\Big|\Big|\sum_{s\in\mathcal{S}}\left(\frac{\exp\{\boldsymbol{U}_{is}^t\boldsymbol{\beta}\}}{1+\sum_{k\in\mathcal{S}}\exp\{\boldsymbol{U}_{ik}^t\boldsymbol{\beta}\}} - \frac{\exp\{\boldsymbol{U}_{is}^t\boldsymbol{\tau}\}}{1+\sum_{k\in\mathcal{S}}\exp\{\boldsymbol{U}_{ik}^t\boldsymbol{\tau}\}}\right)\boldsymbol{U}_{is}\Big|\Big|$$
$$\leq C_1||\boldsymbol{V}_i||^2||\boldsymbol{\beta}-\boldsymbol{\tau}||$$

and by Equation 26, we have that

$$\begin{aligned}\eta_i(\boldsymbol{\tau},\boldsymbol{\beta}) =&\sum_{s\in\mathcal{S}}\left(\frac{\exp\{\boldsymbol{U}_{is}^t\boldsymbol{\beta}\}}{1+\sum_{k\in\mathcal{S}}\exp\{\boldsymbol{U}_{ik}^t\boldsymbol{\beta}\}} - \frac{\exp\{\boldsymbol{U}_{is}^t\boldsymbol{\tau}\}}{1+\sum_{k\in\mathcal{S}}\exp\{\boldsymbol{U}_{ik}^t\boldsymbol{\tau}\}}\right)\boldsymbol{U}_{is}\\ &+E\left[\sum_{s\in\mathcal{S}}\left(\frac{\exp\{\boldsymbol{U}_{is}^t\boldsymbol{\tau}\}}{1+\sum_{k\in\mathcal{S}}\exp\{\boldsymbol{U}_{ik}^t\boldsymbol{\tau}\}} - \frac{\exp\{\boldsymbol{U}_{is}^t\boldsymbol{\beta}\}}{1+\sum_{k\in\mathcal{S}}\exp\{\boldsymbol{U}_{ik}^t\boldsymbol{\beta}\}}\right)\boldsymbol{U}_{is}\right]\end{aligned}$$

So, by Jensen's inequality we have that

$$\begin{aligned}||\eta_i(\boldsymbol{\tau},\boldsymbol{\beta})|| \leq& C_1||\boldsymbol{V}_i||^2||\boldsymbol{\tau}-\boldsymbol{\beta}|| + E\left(C_1||\boldsymbol{V}_i||^2||\boldsymbol{\tau}-\boldsymbol{\beta}||\right)\\ =&C_1\left[||\boldsymbol{V}_i||^2 + E\left(||\boldsymbol{V}_i||^2||\right)\right]||\boldsymbol{\tau}-\boldsymbol{\beta}||\\ \leq& 2C_1M_i||\boldsymbol{\tau}-\boldsymbol{\beta}||.\end{aligned} \tag{29}$$

In Equation 24 of the proof for Sub-Lemma 2.1, we showed that $M_i = O\left(n^{\frac{1}{2}}\right)$. Then, by Example 1 in [1], we have that Condition R1 holds because $M_i = O(n^{r_1})$ for some $r_1 > 0$.

The third condition is

R2. $\sum_{i=1}^n E\left(||\psi((S_i,\boldsymbol{V}_i),\boldsymbol{\beta}_0)||^2\right) = O(nmq)$

Notice that for all $i = 1, 2, ..., n$,

$$\begin{aligned}||\psi((S_i,\boldsymbol{V}_i),\boldsymbol{\beta}_0)|| \leq& \sum_{s\in\mathcal{S}}\left|I(S_i=s) - \frac{\exp\{\boldsymbol{U}_{is}^t\boldsymbol{\beta}_0\}}{1+\sum_{k\in\mathcal{S}}\exp\{\boldsymbol{U}_{ik}^t\boldsymbol{\beta}_0\}}\right| ||\boldsymbol{U}_{i,s}||\\ =&||\boldsymbol{V}_i||\sum_{s\in\mathcal{S}}\left|I(S_i=s) - \frac{\exp\{\boldsymbol{U}_{is}^t\boldsymbol{\beta}_0\}}{1+\sum_{k\in\mathcal{S}}\exp\{\boldsymbol{U}_{ik}^t\boldsymbol{\beta}_0\}}\right|\end{aligned} \tag{30}$$

because $||\boldsymbol{U}_{i,s}|| = ||\boldsymbol{V}_i||$ for all $s \in \mathcal{S}$. Recall that we have also assumed that

$$\begin{aligned}P(S=s|\boldsymbol{X}_i) =& \frac{\exp\{\boldsymbol{U}_{is}^t\boldsymbol{\beta}_0\}}{1+\sum_{k\in\mathcal{S}}\exp\{\boldsymbol{U}_{ik}^t\boldsymbol{\beta}_0\}}\\ P(S=0|\boldsymbol{X}_i) =& \frac{1}{1+\sum_{k\in\mathcal{S}}\exp\{\boldsymbol{U}_{ik}^t\boldsymbol{\beta}_0\}}.\end{aligned}$$

for $s \in \mathcal{S}$ with $\sum_{s\in\{0,\mathcal{S}\}} P(S=s|\boldsymbol{X}_i) = 1$. So, we have that

$$\begin{aligned}\sum_{s\in\mathcal{S}}\left|I(S_i=s) - \frac{\exp\{\boldsymbol{U}_{is}^t\boldsymbol{\beta}_0\}}{1+\sum_{k\in\mathcal{S}}\exp\{\boldsymbol{U}_{ik}^t\boldsymbol{\beta}_0\}}\right| =& \sum_{s\in\mathcal{S}}|I(S_i=s) - P(S=s|\boldsymbol{X}_i)|\\ =&(1-P(S=S_i|\boldsymbol{X}_i)) + \sum_{s\in\mathcal{S};s\neq S_i}P(S=s|\boldsymbol{X}_i)\\ =&(1-P(S=S_i|\boldsymbol{X}_i)) + (1-P(S=S_i|\boldsymbol{X}_i) - P(S=0|\boldsymbol{X}_i))\\ \leq&2\end{aligned} \tag{31}$$

By Equations 30 and 31, we have that

$$||\psi((S_i, \boldsymbol{V}_i), \boldsymbol{\beta}_0)|| \leq 2||\boldsymbol{V}_i||$$
$$\Rightarrow ||\psi((S_i, \boldsymbol{V}_i), \boldsymbol{\beta}_0)||^2 \leq 4||\boldsymbol{V}_i||^2$$

for all $i = 1, 2, ..., n$. So, under Assumption (vii) we have

$$\sum_{i=1}^{n} ||\psi((S_i, \boldsymbol{X}_i), \boldsymbol{\theta}_0)||^2 \leq 4 \sum_{i=1}^{n} |||\boldsymbol{V}_i||^2 = O(nm)$$
$$\Rightarrow \sum_{i=1}^{n} E\left(||\psi((S_i, \boldsymbol{V}_i), \boldsymbol{\beta}_0)||^2\right) = O(nm)$$

and since q is constant $O(nm) = O(nmq)$ and Condition R2 holds

The forth condition is

R3. There exists a sequence of $(mq) \times (mq)$ matrices, $\boldsymbol{D}_n$ with

$$\lim \inf_{n\to\infty} \lambda_{min}(\boldsymbol{D}_n) > 0$$

such that for any $B > 0$ and uniformly in $\boldsymbol{\alpha} \in H_m = \{\boldsymbol{\alpha} \in \mathbb{R}^m : ||\boldsymbol{\alpha}|| = 1\}$

$$\sup_{||\boldsymbol{\beta}-\boldsymbol{\beta}_0|| \leq B\left(\frac{mq}{n}\right)^{\frac{1}{2}}} \left| \boldsymbol{\alpha}^t \sum_{i=1}^{n} E_{\boldsymbol{\beta}_0} \left[\psi((S_i, \boldsymbol{V}_i), \boldsymbol{\beta}) - \psi((S_i, \boldsymbol{V}_i), \boldsymbol{\beta}_0)\right] - n\boldsymbol{\alpha}^t \boldsymbol{D}_n(\boldsymbol{\beta} - \boldsymbol{\beta}_0) \right|$$

is $o((nm)^{\frac{1}{2}})$.

To show Condition R3 holds, note that

$$\psi((S_i, \boldsymbol{V}_i), \boldsymbol{\beta}) = \sum_{s \in \mathcal{S}} \psi_s((S_i, \boldsymbol{V}_i), \boldsymbol{\beta})$$

where each

$$\psi_s((S_i, \boldsymbol{V}_i), \boldsymbol{\theta}) = \left[I(S_i = s) - \frac{\exp\{\boldsymbol{U}_{is}^t \boldsymbol{\beta}\}}{1 + \sum_{k \in \mathcal{S}} \exp\{\boldsymbol{U}_{ik}^t \boldsymbol{\beta}\}} \right] \boldsymbol{U}_{is}$$

From Example 3 in He and Shao [1], we have that

$$\left| \boldsymbol{\alpha}^t \sum_{i=1}^{n} E_{\boldsymbol{\beta}_0} \left[\psi_s((S_i, \boldsymbol{V}_i), \boldsymbol{\beta}) - \psi_s((S_i, \boldsymbol{V}_i), \boldsymbol{\beta}_0)\right] - n\boldsymbol{\alpha}^t \boldsymbol{G}_{n,s}(\boldsymbol{\beta} - \boldsymbol{\beta}_0) \right|$$
$$\leq (\boldsymbol{\beta} - \boldsymbol{\beta}_0)^t \sum_{i=1}^{n} |\boldsymbol{\alpha}^t \boldsymbol{U}_{is}| \boldsymbol{U}_{is} \boldsymbol{U}_{is}^t (\boldsymbol{\beta} - \boldsymbol{\beta}_0)$$

where

$$\boldsymbol{G}_{n,s} = \frac{1}{n} \sum_{i=1}^{n} \frac{\exp\{\boldsymbol{U}_{is}^t \boldsymbol{\beta}\}}{1 + \sum_{k \in \mathcal{S}} \exp\{\boldsymbol{U}_{ik}^t\}} \boldsymbol{U}_{i,s} \boldsymbol{U}_{i,s}^t.$$

Because $\boldsymbol{G}_n$ as defined in Condition (xi) is $\sum_{s\in\mathcal{S}} \boldsymbol{G}_{n,s}$, we have that

$$\begin{aligned}
&\left|\boldsymbol{\alpha}^t \sum_{i=1}^{n} E_{\boldsymbol{\beta}_0}\left[\psi((S_i,\boldsymbol{V}_i),\boldsymbol{\beta}) - \psi((S_i,\boldsymbol{V}_i),\boldsymbol{\beta}_0)\right] - n\boldsymbol{\alpha}^t\boldsymbol{G}_n(\boldsymbol{\beta}-\boldsymbol{\beta}_0)\right| \\
&\quad \leq \sum_{s\in\mathcal{S}} \left|\boldsymbol{\alpha}^t \sum_{i=1}^{n} E_{\boldsymbol{\beta}_0}\left[\psi_s((S_i,\boldsymbol{V}_i),\boldsymbol{\beta}) - \psi_s((S_i,\boldsymbol{V}_i),\boldsymbol{\beta}_0)\right] - n\boldsymbol{\alpha}^t\boldsymbol{G}_{n,s}(\boldsymbol{\beta}-\boldsymbol{\beta}_0)\right| \\
&\quad \leq (\boldsymbol{\beta}-\boldsymbol{\beta})^t \sum_{i=1}^{n}\sum_{s\in\mathcal{S}} |\boldsymbol{\alpha}^t\boldsymbol{U}_{is}|\boldsymbol{U}_{is}\boldsymbol{U}_{is}^t(\boldsymbol{\beta}-\boldsymbol{\beta}) \\
&\quad = \sum_{i=1}^{n}\sum_{s\in\mathcal{S}} |\boldsymbol{\alpha}^t\boldsymbol{U}_{is}| \left[\boldsymbol{U}_{is}^t(\boldsymbol{\beta}-\boldsymbol{\beta})\right]^2
\end{aligned}$$

By Condition (ix) we have that

$$\sup_{\boldsymbol{\alpha},\boldsymbol{\gamma}\in H_{qm}} \sum_{i=1}^{n}\sum_{s\in\mathcal{S}} (\boldsymbol{\alpha}^t\boldsymbol{U}_{is})^2(\boldsymbol{\gamma}^t\boldsymbol{U}_{is})^2 \leq \sum_{s\in\mathcal{S}} \left[\sup_{\boldsymbol{\alpha},\boldsymbol{\gamma}\in H_{pm}} \sum_{i=1}^{n} (\boldsymbol{\alpha}^t\boldsymbol{U}_{is})^2(\boldsymbol{\gamma}^t\boldsymbol{U}_{is})^2\right] = O(nm)$$

Then, by results from Example 1 in [1], we have that

$$\sup_{||\boldsymbol{\beta}-\boldsymbol{\beta}_0||\leq B\left(\frac{mp}{n}\right)^{\frac{1}{2}}} \left|\boldsymbol{\alpha}^t \sum_{i=1}^{n} E_{\boldsymbol{\beta}_0}\left[\psi((S_i,\boldsymbol{V}_i),\boldsymbol{\beta}) - \psi((S_i,\boldsymbol{V}_i),\boldsymbol{\beta}_0)\right] - n\boldsymbol{\alpha}^t\boldsymbol{G}_n(\boldsymbol{\beta}-\boldsymbol{\beta}_0)\right|$$

is $o((nm)^{\frac{1}{2}})$ and Condition R3 holds.

The fifth and sixth conditions are

R4. For any $\boldsymbol{\beta} \in \mathbb{R}^{mq}$, $\boldsymbol{\alpha} \in H_{qm}$, and $B > 0$,

$$\sup_{\boldsymbol{\tau}:||\boldsymbol{\tau}-\boldsymbol{\theta}||\leq B\left(\frac{qm}{n}\right)^{\frac{1}{2}}} \left\{\sum_{i=1}^{n} E_{\boldsymbol{\theta}}\left[|\boldsymbol{\alpha}^t\eta_i(\boldsymbol{\tau},\boldsymbol{\beta})|^2\right]\right\} = O(m)$$

R5. For any $\boldsymbol{\beta} \in \mathbb{R}^{mq}$ and $B > 0$,

$$\sup_{\boldsymbol{\alpha}\in H_{qm}} \left\{\sup_{\boldsymbol{\tau}:||\boldsymbol{\tau}-\boldsymbol{\beta}||\leq B\left(\frac{qm}{n}\right)^{\frac{1}{2}}} \sum_{i=1}^{n} \left[\boldsymbol{\alpha}^t\eta_i(\boldsymbol{\tau},\boldsymbol{\theta})\right]^2\right\} = O_P(m)$$

and follow from arguments very similar to those used in the proof for for Sub-Lemma 2.1 based on the bound on $||\eta_i(\boldsymbol{\tau},\boldsymbol{\beta})||$ established in Equation 29.

□

2 Additional Simulation Details

As a reminder, the general simulation settings we considered are:

Setting	Study Sizes	Outcome Coefficients	Main Source of Treatment Effect Heterogeneity
1	Similar	Random	Measured Features
2	Different	Random	Measured Features
3	Different	Random	Unmeasured Features

2.1 Approximate Study Sizes

We used a numeric algorithm to solve for study-specific intercepts that resulted in, on average, specified target population sample and study sample sizes [2]. When studies were relatively large and of similar sizes, intercepts were generated that resulted in, on average, a target population sample of around 5,500 participants and studies of around 1,500 participants. The target population sample was instead around 200 participants and studies around 100 participants in settings where studies were small and and of similar sizes.

In setting where we had studies of different sizes, the target population samples were again around 5,500 participants in settings where studies were large and around 200 participants in settings where studies were small. See Table 1 for the approximate study sizes. These study sizes

Number of Studies (m)	Large or Small Studies	Average Study Sizes (Study 1 to Study m)
3	Small	41, 80, 179
3	Large	675, 1350, 2475
10	Small	33, 45, 81, 83, 105, 108, 114, 133, 142, 157
10	Large	754, 778, 962, 1229, 1417, 1629, 1678, 2057, 2172, 2324
30	Small	40, 52, 53, 54, 54, 55, 55, 58, 63, 74, 74, 82, 88, 91, 107, 107, 110, 114, 117, 120, 122, 126, 129, 136, 143, 144, 152, 159, 160, 161
30	Large	265, 271, 287, 377, 478, 557, 596, 693, 695, 766, 810, 829, 899, 987, 1450, 1472, 1730, 2023, 2122, 2160, 2161, 2194, 2433, 2433, 2436, 2575, 2619, 2830, 2865, 2988

Table 1: Approximate Study Sizes for Simulation with Studies of Different Sizes

were generated by sampling m values from a Unif$(0, 1)$ distribution, dividing each by the sum of the values (so they sum to one), sorting them from smallest to largest, and assigning these to be the marginal probabilities of study membership conditional on not being in the target population sample for Studies 1 to m .

2.2 Coefficients for Study Membership Model

In our simulations, observations were assigned to either a study $s \in \mathcal{S} = \{1, 2, ..., m\}$ or membership in the target population using a multinomial logistic regression model where

$$P(S_i = s|X_i) = \text{expit}\left(\beta_{s,0} + X_i\beta_{s,1}\right), \text{ for } s = 1, 2, ..., m$$
$$P(S_i = 0|X_i) = 1 - \sum_{s=1}^{m} P(S_i = s|X_i).$$

The following tables contain the values of $\beta_{s,0}$ and $\beta_{s,1}$, $s = 1, 2, ..., m$ used in each setting.

Setting	Large or Small Studies	$\beta_{s,0}$ from $1, .., m$
1	Small	-1.38, -1.31, -1.30
1	Large	-1.347, -1.302, -1.299
2 and 3	Small	-1.66, -0.94, -0.11
2 and 3	Large	-2.16, -1.414, -0.799

Table 2: Values of $\beta_{s,0}$ in each setting when $m = 3$. Coefficients are listed in order from Study 1 to Study m.

Setting	Large or Small Studies	$\beta_{s,0}$ from $1, .., m$
1, 2, and 3	Small	-0.40 -0.19, 0
1, 2, and 3	Large	-0.40, -0.19, 0

Table 3: Values of $\beta_{s,1}$ in each setting when $m = 3$. Coefficients are listed in order from Study 1 to Study m.

Setting	Large or Small Studies	$\beta_{s,0}$ from $1, .., m$
1	Small	-0.71, -0.70, -0.69, -0.68, -0.68,<br>-0.68, -0.69, -0.69, -0.69, -0.69
1	Large	-1.32, -1.31, -1.30, -1.29, -1.29,<br>-1.29, -1.29, -1.29, -1.29, -1.30
2 and 3	Small	-1.84, -1.51, -0.92, -0.87, -0.64,<br>-0.62, -0.56, -0.41, -0.34, -0.24
2 and 3	Large	-2.02, -1.97, -1.75, -1.49, -1.35,<br>-1.21, -1.18 -0.98 -0.92, -0.86

Table 4: Values of $\beta_{s,0}$ in each setting when $m = 10$. Coefficients are listed in order from Study 1 to Study m.

Setting	Large or Small Studies	$\beta_{s,0}$ from $1, .., m$
1, 2, and 3	Small	-0.39, -0.33, -0.26, -0.19, -0.15,<br>-0.12, -0.03, -0.03, -0.02, -0.02
1, 2, and 3	Large	-0.39, -0.32, -0.28, -0.18, -0.17,<br>-0.16, -0.16, -0.09, -0.05, -0.01

Table 5: Values of $\beta_{s,1}$ in each setting when $m = 10$. Coefficients are listed in order from Study 1 to Study m.

Setting	Large or Small Studies	$\beta_{s,0}$ from $1,..,m$
1	Small	-0.69, -0.69, -0.69, -0.69, -0.69, -0.68, -0.68, -0.68, -0.68, -0.68, -0.68, -0.68, -0.68, -0.67, -0.67, -0.68, -0.68, -0.68, -0.68, -0.68, -0.68, -0.68, -0.68, -0.68, -0.68, -0.69, -0.69, -0.69, -0.69, -0.69
1	Large	-1.304, -1.302, -1.302, -1.301, -1.3, -1.295, -1.294, -1.294, -1.294, -1.293, -1.293, -1.29, -1.289, -1.288, -1.287, -1.286, -1.286, -1.286, -1.286, -1.287, -1.287, -1.288, -1.289, -1.29, -1.291, -1.291, -1.293, -1.296, -1.297, -1.299
2 and 3	Small	-1.63, -1.36, -1.34, -1.32, -1.32, -1.29, -1.28, -1.24, -1.16, -1.00, -0.99, -0.88, -0.81, -0.77, -0.62, -0.61, -0.58, -0.55, -0.53, -0.50, -0.49, -0.45, -0.43, -0.38, -0.33, -0.32, -0.27, -0.22, -0.22, -0.21
2 and 3	Large	-3.058, -3.033, -2.976, -2.7, -2.463, -2.303, -2.235, -2.084, -2.08, -1.982, -1.925, -1.897, -1.815, -1.719, -1.333, -1.315, -1.152, -0.995, -0.948, -0.93, -0.929, -0.913, -0.811, -0.811, -0.81, -0.754, -0.738, -0.663, -0.65, -0.61

Table 6: Values of $\beta_{s,0}$ in each setting when $m = 30$. Coefficients are listed in order from Study 1 to Study m.

Setting	Large or Small Studies	$\beta_{s,0}$ from $1, .., m$
1, 2, and 3	Small	-0.38, -0.37, -0.37, -0.33, -0.33, -0.32, -0.31, -0.28, -0.28, -0.26, -0.25, -0.24, -0.22, -0.19, -0.19, -0.17, -0.15, -0.15, -0.15, -0.14, -0.12, -0.10, -0.09, -0.07, -0.05, -0.04, -0.04, -0.03, -0.02, -0.02
1, 2, and 3	Large	-0.35, -0.34, -0.34, -0.33, -0.33, -0.29, -0.29, -0.29, -0.28, -0.28, -0.28, -0.24, -0.24, -0.22, -0.21, -0.17, -0.15, -0.14, -0.14, -0.13, -0.13, -0.10, -0.08, -0.07, -0.07, -0.07, -0.04, -0.02, -0.02, 0.00

Table 7: Values of $\beta_{s,1}$ in each setting when $m = 10$. Coefficients are listed in order from Study 1 to Study m.

2.3 TATEs Used to Evaluate Performance

In order to evaluate the performance of the estimators, we had to be able to calculate the true TATE in the target population. This requires knowing $E(X|S = 0)$. To obtain a close approximation to this value, we conducted simulations with 50,000 replications. The resulting approximations to the true TATEs, as well as the approximations to $E(X|S = 0)$ used to obtain them, in each setting are:

Small or Large Studies	m	Settings	TATE	$E(X\|S = 0)$	MCSE of $E(X\|S = 0)$
Small	3	1	-1.04	0.08	0.00027
		2 and 3	-1.03	0.06	0.00031
	10	1	-1.06	0.13	0.00032
		2 and 3	-1.04	0.09	0.00031
	30	1	-1.09	0.18	0.00032
		2 and 3	-1.07	0.14	0.00032
Large	3	1	-1.04	0.09	5.9×10^{-5}
		2 and 3	-1.03	0.05	5.9×10^{-5}
	10	1	-1.06	0.13	6×10^{-5}
		2 and 3	-1.05	0.10	6×10^{-5}
	30	1	-1.08	0.17	6×10^{-5}
		2 and 3	-1.05	0.11	6×10^{-5}

2.4 Full Simulation Results

Three Large Studies

Metric	Estimator	Setting 1	Setting 2	Setting 3
Bias	Unadjusted	0.091 (0.0080)	0.056 (0.0074)	0.099 (0.0286)
	Pooled	**-0.007 (0.0077)**	**-0.002 (0.0079)**	**0.040 (0.0287)**
	Two-Stage	**-0.003 (0.0062)**	**-0.006 (0.0061)**	**0.035 (0.0256)**
EmpSE	Unadjusted	0.252 (0.0056)	0.235 (0.0053)	0.903 (0.0202)
	Pooled	0.242 (0.0054)	0.249 (0.0056)	0.906 (0.0203)
	Two-Stage	**0.195 (0.0044)**	**0.194 (0.0043)**	**0.809 (0.0181)**
MSE	Unadjusted	0.071 (0.0032)	0.058 (0.0025)	0.824 (0.0359)
	Pooled	0.059 (0.0025)	0.062 (0.0028)	0.822 (0.036)
	Two-Stage	**0.038 (0.0016)**	**0.038 (0.0017)**	**0.655 (0.0307)**

Ten Large Studies

	Estimator	Setting 1	Setting 2	Setting 3
Bias	Unadjusted	0.089 (0.0037)	0.075 (0.0038)	0.083 (0.0154)
	Pooled	**-0.0001 (0.0041)**	**0.005 (0.0042)**	**0.015 (0.0154)**
	Two-Stage	**-0.0007 (0.0037)**	**0.003 (0.0036)**	**0.015 (0.0145)**
Emp. SE	Unadjusted	0.115 (0.0026)	0.119 (0.0027)	0.486 (0.0109)
	Pooled	0.129 (0.0029)	0.133 (0.003)	0.486 (0.0109)
	Two-Stage	**0.118 (0.0026)**	**0.113 (0.0025)**	**0.457 (0.0102)**
MSE	Unadjusted	0.021 (0.0009)	0.020 (0.0009)	0.243 (0.0106)
	Pooled	0.017 (0.0007)	0.018 (0.0008)	0.236 (0.0102)
	Two-Stage	**0.014 (0.0006)**	**0.013 (0.0006)**	**0.209 (0.0092)**

Thirty Large Studies

	Estimator	Setting 1	Setting 2	Setting 3
Bias	Unadjusted	0.094 (0.0021)	0.058 (0.0023)	0.053 (0.0094)
	Pooled	**0.002 (0.0025)**	**-0.003 (0.0027)**	**-0.007 (0.0095)**
	Two-Stage	**0.003 (0.0023)**	**-0.004 (0.0021)**	**-0.008 (0.0079)**
Emp. SE	Unadjusted	0.066 (0.0015)	0.073 (0.0016)	0.298 (0.0067)
	Pooled	0.079 (0.0018)	0.085 (0.0019)	0.299 (0.0067)
	Two-Stage	**0.073 (0.0016)**	**0.067 (0.0015)**	**0.250 (0.0056)**
MSE	Unadjusted	0.013 (0.0004)	0.009 (0.0004)	0.092 (0.0039)
	Pooled	0.006 (0.0003)	0.007 (0.0003)	0.089 (0.0038)
	Two-Stage	**0.005 (0.0002)**	**0.005 (0.0002)**	**0.063 (0.0029)**

Table 8: Results from simulations with three, ten, and thirty large studies; simulations had 1,000 replications; Monte Carlo standard errors are shown in parentheses; lowest values in for each criteria for each setting are highlighted with bold font

Three Small Studies

	Estimator	Setting 1	Setting 2	Setting 3
Bias	Unadjusted	0.086 (0.0103)	0.0429 (0.0097)	0.0404 (0.0298)
	Pooled	**-0.015 (0.0092)**	**0.001 (0.0095)**	**-0.013 (0.0298)**
	Two-Stage	**-0.014 (0.0082)**	**0.003 (0.0087)**	**-0.007 (0.0261)**
EmpSE	Unadjusted	0.326 (0.0073)	0.308 (0.0069)	0.942 (0.0211)
	Pooled	0.291 (0.0065)	0.300 (0.0067)	0.942 (0.0211)
	Two-Stage	**0.259 (0.0058)**	**0.275 (0.0061)**	**0.827 (0.0185)**
MSE	Unadjusted	0.114 (0.0053)	0.097 (0.0045)	0.887 (0.0423)
	Pooled	0.085 (0.0041)	0.090 (0.004)	0.886 (0.0422)
	Two-Stage	**0.067 (0.0031)**	**0.075 (0.0037)**	**0.683 (0.0321)**

Ten Small Studies

	Estimator	Setting 1	Setting 2	Setting 3
Bias	Unadjusted	0.077 (0.0050)	0.047 (0.0050)	0.056 (0.0156)
	Pooled	**0.002 (0.0050)**	**-0.004 (0.0049)**	**0.002 (0.0155)**
	Two-Stage	**0.003 (0.0045)**	**-0.002 (0.0045)**	**0.006 (0.0144)**
Emp. SE	Unadjusted	0.157 (0.0035)	0.157 (0.0035)	0.492 (0.0110)
	Pooled	0.158 (0.0035)	0.155 (0.0035)	0.491 (0.011)
	Two-Stage	**0.143 (0.0032)**	**0.142 (0.0032)**	**0.456 (0.0102)**
MSE	Unadjusted	0.031 (0.0014)	0.027 (0.0012)	0.245 (0.0112)
	Pooled	0.025 (0.0012)	0.024 (0.0011)	0.241 (0.0112)
	Two-Stage	**0.021 (0.0010)**	**0.020 (0.0009)**	**0.208 (0.0095)**

Thirty Small Studies

	Estimator	Setting 1	Setting 2	Setting 3
Bias	Unadjusted	0.0958 (0.0029)	0.0705 (0.003)	0.0759 (0.0089)
	Pooled	**0.002 (0.0032)**	**-0.001 (0.0033)**	**0.004 (0.0088)**
	Two-Stage	**0.002 (0.0029)**	**-0.001 (0.0030)**	**0.002 (0.0083)**
Emp. SE	Unadjusted	**0.093 (0.0021)**	**0.094 (0.0021)**	**0.280 (0.0063)**
	Pooled	0.101 (0.0023)	0.105 (0.0023)	**0.279 (0.0062)**
	Two-Stage	**0.091 (0.002)**	**0.096 (0.0021)**	**0.261 (0.0058)**
MSE	Unadjusted	0.018 (0.0007)	0.014 (0.0006)	0.084 (0.0039)
	Pooled	0.010 (0.0005)	0.011 (0.0005)	0.078 (0.0035)
	Two-Stage	**0.008 (0.0004)**	**0.009 (0.0004)**	**0.068 (0.0030)**

Table 9: Results from simulations with three, ten, and thirty small studies; simulations had 1,000 replications; Monte Carlo standard errors are shown in parentheses; lowest values in for each criteria for each setting are highlighted with bold font

Table 10: Coverage of the 95% percentile intervals and the relative error in the bootstrapped standard error estimates derived using proposed stratified bootstrap procedure for simulations under Setting 2 with large study sizes; Simulations with $m = 3$ and $m = 10$ studies had 1,000 replications; Simulations with $m = 30$ studies had 500 replications; Bootstrapped estimates were derived using 1,000 bootstrap samples; Monte Carlo standard errors are shown in parentheses

	Estimator	Three Large Studies	Ten Large Studies	Thirty Large Studies
Coverage	Unadjusted	0.772 (0.0133)	0.863 (0.0109)	0.826 (0.017)
	Pooled	0.813 (0.0123)	0.916 (0.0088)	0.970 (0.0076)
	Two-Stage	0.761 (0.0135)	0.915 (0.0088)	0.962 (0.0086)
Rel. Error	Unadjusted	-5.16% (2.97)	2.81% (2.44)	17.9% (3.81)
	Pooled	-28.3% (2.07)	-10.4% (2.13)	5.30% (3.40)
	Two-Stage	-25.3% (2.22)	-4.75% (2.25)	7.20% (3.44)

Table 11: Coverage of the 95% percentile intervals and the relative error in the bootstrapped standard error estimates derived using proposed stratified bootstrap procedure for simulations under Setting 2 with small study sizes; Simulations with $m = 3$ and $m = 10$ studies had 1,000 replications; Simulations with $m = 30$ studies had 500 replications; Bootstrapped estimates were derived using 1,000 bootstrap samples; Monte Carlo standard errors are shown in parentheses

	Estimator	Three Small Studies	Ten Small Studies	Thirty Small Studies
Coverage	Unadjusted	0.907 (0.0092)	0.959 (0.0063)	0.902 (0.0133)
	Pooled	0.914 (0.0089)	0.970 (0.0054)	0.980 (0.0063)
	Two-Stage	0.927 (0.0082)	0.970 (0.0054)	0.980 (0.0063)
Rel. Error	Unadjusted	10.4% (3.15)	27.7% (3.00)	28.6% (4.14)
	Pooled	-3.66% (2.51)	14.7% (2.68)	16.3% (3.76)
	Two-Stage	-0.91% (2.49)	13.0% (2.65)	22.1% (3.95)

References

[1] Xuming He and Qi-Man Shao. On parameters of increasing dimensions. *Journal of Multivariate Analysis*, 73(1):120–135, 2000.

[2] Sarah E Robertson, Jon A Steingrimsson, and Issa J Dahabreh. Using numerical methods to design simulations: Revisiting the balancing intercept. *American Journal of Epidemiology*, 191(7):1283–1289, 2022.
